# Exploration of spatial heterogeneity of tumor microenvironment in nasopharyngeal carcinoma via transcriptional digital spatial profiling

**DOI:** 10.7150/ijbs.74653

**Published:** 2023-04-17

**Authors:** Liping Wang, Dujuan Wang, Xiaojiao Zeng, Qian Zhang, Huiqing Wu, Jie Liu, Yang Wang, Guohong Liu, Yunbao Pan

**Affiliations:** 1 Department of Laboratory Medicine, Zhongnan Hospital of Wuhan University, Wuhan University, Wuhan, Hubei, China.; 2 Department of Clinical Pathology, Houjie Hospital of Dongguan, The Affiliated Houjie Hospital of Guangdong Medical University, Dongguan, China.; 3 Department of Radiology, Zhongnan Hospital of Wuhan University, Wuhan University, Wuhan, China.; 4 State Key Laboratory of Biocatalysis and Enzyme Engineering, School of Life Sciences, Hubei University, Wuhan, China.

**Keywords:** Nasopharyngeal carcinoma, Tumor microenvironment, Immune infiltration, Metabolic marker, Immune checkpoints, Digital spatial profiling

## Abstract

The heterogeneity of nasopharyngeal carcinoma (NPC) leads to mixed clinical outcomes. We collected 92 regions of interest from 41 biopsies of patients with untreated NPC and obtained their transcripts using GeoMx Digital Spatial Profiling (DSP) technology. Spatial heterogeneity was determined by measuring the expression of marker genes in tumor cell-enriched (PanCK-expressing), immune cell-enriched (CD45-expressing), and normal epithelial (Endo) regions. We screened 16 prognostic markers in tumor cell-enriched regions and 4 prognostic markers in immune cell-enriched regions. The levels of CD8^+^ T follicular helper T cells, activated NK cells, and M0 macrophage contents were higher in tumor cell-enriched regions than in immune cell-enriched regions. Conversely, plasma cell and M2 macrophage levels were lower. The follicular helper T cells in tumor cell-enriched regions were negatively correlated with resting NK cells and positively correlated with activated NK cells. In immune cell-enriched regions, this relationship was reversed. We also explored the heterogeneity of HLA gene families, immune checkpoints, and metabolism-related genes in the three regions. In tumor cell-enriched regions, we obtained 19 prognosis-related metabolism genes via univariate cox analysis. We used multiplex immunofluorescence to verify the elevated expression of SLC8A1 and MDH1 in immune cell-enriched regions and tumor cell-enriched regions, respectively, both of which were associated with prognosis of NPC. In conclusion, we explored the spatial heterogeneity of the NPC tumor environment and found specific diagnostic and prognostic markers that can be used to differentiate tumor cell-enriched regions from immune cell-enriched regions in NPC.

## Introduction

Nasopharyngeal carcinoma (NPC) is mainly prevalent in North Africa, the Middle East, and Southeast Asia. Its occurrence is mainly attributed to the local environment, genetics, and infection with Epstein-Barr virus (EBV) 1. In 2018, approximately 130,000 cases were confirmed and over 70,000 deaths of NPC were recorded globally. In 2020, more than 130,000 new cases of NPC and 80,000 deaths occurred worldwide. These numbers show a consistently high prevalence and increasing mortality rate due to the disease 2. Approximately 85% of these patients reside in Asia, and those at high risk of developing NPC are typically diagnosed at 45-59 years of age 3. Radiotherapy and immune-targeted therapy are the two common clinical treatments for NPC, each with its own drawbacks. Although radiotherapy has been partly successful in treating NPC, its side effects are not well tolerated. As for immune-targeted therapy, the heterogeneity of the tumor microenvironment (TME) in NPC patients can lead to uneven outcomes. The disease burden may be lowered by determining the interactions among components of the NPC microenvironment and developing biomarkers for earlier diagnosis 4.

The tumor microenvironment is composed of tumor cells, immune cells, stromal cells, metabolites, cytokines, and the extracellular matrix 5, 6. It is highly heterogeneous and complex in different patients with NPC 7. Different regions show distinct cellular composition, active metabolic pathways, and overall function 8. The role of cells and the extracellular matrix at these different locations remain unknown, suggesting the need to establish region-specific biomarkers for clinical diagnosis and therapy.

The current study explored the spatial heterogeneity of NPCs in both composition and function. The digital spatial profiling (DSP) technology 9 was used to measure immune cell fractions, immune checkpoints, immune function, activities of HLA genes, immune cell functions impacting survival, and prognostic biomarkers in tumor cells (PanCK-expressing) and in immune cells areas (CD45-expressing). We also extracted metabolism-related genes, explored the signaling pathways involving these genes at different regions, and screened for prognosis-related metabolic markers in tumor cells areas.

## Methods

### Patient sample collection

We collected paraffin-embedded tumor tissue from 58 NPC patients who underwent initial nasopharyngeal biopsy without treatment at the Affiliated Houjie Hospital of Guangdong Medical University. This study was approved by the Ethics and Scientific Committee of the Affiliated Houjie Hospital of Guangdong Medical University. A tissue microarray (TMA) was constructed with 1.5 mm^2^ tissue cores obtained from 58 patients using a TMArrayer (Pathology Devices). Regions consisting of high tumor content with large numbers of tumor infiltrating lymphocytes (TILS) were selected following preparation of the TMA block using H&E sections from the whole tissue block to ensure the presence of NPC cells in each sample. Subsequent DSP RNA assays were performed using 41 cores including 95 regions of interest (41 pairs of regions enriched with tumor and immune cells, and 13 regions enriched with normal epithelial cells). Three regions of interest (ROIs) were excluded from paired analyses due to loss of tissue integrity during staining. Finally, 38 cores containing paired tumor cell- and immune cell-enriched ROIs were included in the differential analysis.

Another commercial NPC TAM (Shanghai Mingyi Biotechnology Co., Ltd) containing 126 cases of NPC tissue were used for Immunofluorescence analysis. Five cores were excluded from analyses due to loss of tissue integrity during staining. Extreme values (< 10th percentile; > 90th percentile) were excluded from survival analysis.

### Digital spatial profiling and analysis

NanoString GeoMx DSP RNA assays were performed at CapitalBio Technology (Beijing, China) using the standard protocol. Slides were prepared following the Manual RNA Slide Preparation Protocol in the GeoMx DSP Slide Preparation User Manual (NanoString, MAN-10115-05 for software v2.3). The Whole Transcriptome Atlas (WTA) probe reagent was added to slide. We stained the tissue with GeoMx Solid Tumor TME Morphology Kit (Nanostring, Cat#GMX-RNA-MORPH-HST-12) to distinguish various morphology: epithelial cells were positively stained with PanCK while immune cells were positively stained with CD45, and nuclear stained with SYTO13. The pathologist distinguishes normal epithelial cells from cancerous epithelial cells based on histological morphology. Regions of interest (ROIs) were selected and assessed by a pathologist and illuminated using UV light. The indexing oligonucleotides released from each ROI were collected and deposited into designated wells on a microtiter plate. DSP assay sequencing data were processed with the GeoMx NGS Pipeline (DND). After sequencing, the reads were trimmed, merged, and aligned to a list of indexing oligos to identify the source probe. The unique molecular identifier (UMI) region of each read was used to remove PCR duplicates and duplicate reads, thus converting reads into digital counts. The limit of quantitation (LOQ) was estimated as the geometric mean of the negative control probes plus two geometric standard deviations of the negative control probes. Targets that consistently fell below the LOQ were removed, and the datasets were normalized via upper quartile (Q3) normalization. We used the prcomp function to perform principal component analysis from the gene expression matrix and plotted it with the scatterplot3d package.

### Differential expression and enrichment analysis

Comparisons between the two groups were performed using the Mann-Whitney U test or Wilcoxon signed-ranks test. Genes of significance were defined based on a fold change > 1.5 and p-values <0.05. Gene ontology (GO) enrichment and KEGG enrichment analyses of DEGs were performed using clusterProfiler R-packages with Benjamini-Hochberg multiple testing adjustment.

### Weighted correlation network analysis (WGCNA) and protein-protein interaction (PPI) networks of differentially expressed genes

Weighted correlation network analysis (WGCNA) of 2940 differential genes filtered from 18675 genes from matched 38 PanCK-expressing and 38 CD45-expressing regions was performed using the WGCNA and limma packages. The gene expression matrix was converted into a Pearson correlation coefficient matrix. The adjacency matrix was constructed based on the optimal power value (power = 6, Scale Free Topology R^2^ =0.89) for the Pearson correlation coefficient matrix. A topology overlap matrix (TOM) was constructed to perform hierarchical clustering with at least 50 genes per module (minClusterSize =50). The cutreeDynamic function was used to automatically cut the clustered modules and merge similar modules (abline = 0.25). The turquoise module contained 569 genes. The green module carried 267 genes and the blue module included 1010 genes. The genes of modules were extracted for GO (q =0.05) and KEGG (p =0.05) functional enrichment analysis. The R packages involved in these analyses were: clusterProfiler, org.Hs.eg.db, enrichplot, and ggplot2. Genes from the turquoise module (n=569), green module (n=267) and blue module (n=1010) were respectively submitted to the STRING database (http://www.string-db.org/). Parameter settings were as follows: network type (full STRING network), meaning of network edges (confidence), and minimum required interaction score (high confidence (0.700). Protein interaction data obtained from the STRING database were entered into the Cytoscape software. The cytoHubba plugin was used to screen core genes in the PPI network.

### Prognostic gene screening

The blue module (n=1010) obtained from 38 PanCK-expressing regions was screened for 16 prognosis-related genes by univariate Cox analysis. The optimal cutoff value of each of these genes was determined according to the surv_cutpoint and surv_categorize functions. The expression of each gene was grouped according to the optimal cutoff value, followed by survival analysis with the log-rank test. The turquoise module (n=569) of 38 CD45-expressing regions was screened for four prognosis-related genes by univariate Cox analysis. The optimal cutoff value for each of these four genes was determined according to the surv_cutpoint and surv_categorize functions. The expression of each gene was categorized according to the optimal cutoff value, followed by survival analysis with the log-rank test. The R packages involved in the above process were survival and survminer.

### Immune infiltration analysis

The relative content of 22 immune cells was calculated for each of the 92 ROIs (40 PanCK-expressing, 39 CD45-expressing, and 13 Endo) using the CIBERSORT algorithm. Immune cells were visualized in 90 ROIs (39 PanCK-expressing, 38 CD45-expressing, and 13 Endo) at P < 0.05. Differences in immune cell levels among the three regions (39 PanCK-expressing, 38 CD45-expressing, and 13 Endo) were identified using the Kruskal-Wallis test with the reshape2 and ggpubr packages. A set of genes related to immune function was obtained based on previous studies 10. Single-sample Gene Set Enrichment Analysis (GSEA) was performed using the packages GSVA, limma, and GSEABase based on the immune function-related gene set to obtain the corresponding immune function scores for each ROI (92 ROIs in total). The scores among the three regions were visualized via the Wilcoxon test using the limma, ggplot2, and ggpubr packages. A correlation analysis of the immune cell contents of PanCK-expressing and CD45-expressing regions was performed separately using the corrplot package with the Spearman method. The cutoff values of 22 immune cell contents in PanCK-expressing regions were obtained using the surv_cutpoint and surv_categorize functions, and then grouped according to the cutoff values. Survival analysis of immune cells in PanCK-expressing regions was performed using the limma, survival, and survminer packages with the log-rank test (P <0.05). Similarly, the immune function scores were grouped according to cutoff values and the survival analyses of immune function scores in PanCK- and CD45-expressing regions were performed using the limma, survival, and survminer packages with the log-rank test (P <0.05).

### HLA expression and immune checkpoint analysis

Differential expression of HLA genes in the three regions (92 ROIs) was determined using the limma, reshape2, ggplot2, and ggpubr packages via Kruskal-Wallis test. Differential expression of immune checkpoint-related genes in the three regions was explored using limma, ggplot2 and ggpubr packages via Wilcoxon test. Correlation analysis between immune checkpoint gene expression and immune cell levels was performed in PanCK- and CD45-expressing regions using the limma, reshape2, tidyverse, and ggplot2 packages with Spearman correlation coefficients. This analysis was visualized.

### Analysis of metabolism-related genes

We obtained 944 genes related to metabolism based on the literature 11. The file "c2.cp.kegg.v7.5.1.symbols.gmt" was obtained from the Gene Set Enrichment Analysis (GSEA) website (http://www.gsea-msigdb.org/gsea/downloads.jsp). GSVA of metabolism-related genes was performed in 92 ROIs using the packages GSEABase, GSVA, limma, and pheatmap. Parameters were set to P < 0.05 and only 20 pathways were shown. A total of 134 genes were obtained by intersecting 944 metabolic genes with 2940 differential genes (PanCK- vs CD45-expressing regions) and plotting the Venn diagram based on the Bioinformatics (http://bioinformatics.psb.ugent.be/webtools/Venn/) website. Univariate Cox analysis (P < 0.05) of 134 genes was performed in 38 (PanCK-expressing) ROIs to screen for prognosis-related metabolic genes, survival analysis, and ROC curve plotting. A total of 171 genes were obtained by intersecting 944 metabolic genes with 2514 differential genes (PanCK-expressing vs Endo regions). Univariate cox analysis, survival analysis, and ROC curve plotting of 171 genes were performed in 38 (PanCK-expressing) ROIs. The R packages involved in univariate cox analysis, survival analysis, and ROC curve plotting were survivor, survminer, and timeROC.

### Multiplex immunofluorescence staining

TMA section was deparaffinized to retrieve the antigen and block endogenous peroxidase, according to manufacturer's instructions. Tissue sections were blocked with 3% BSA in TBST for 30 min, and then incubated with the antibody for CD45 (ZSGB-BIO, Cat#ZM-0183) for 30 min. The antibody was detected using the corresponding secondary antibody tagged with HRP, before visualization using CY3-TSA. Subsequently, antigen was retrieved again to prepare the slides for the next antibody. All samples were stained sequentially with CK (ZSGB-BIO, Cat#ZM0069) visualized with FITC-TSA, MDH1 (Proteintech, Cat#15904-1-AP) visualized with Opal 647 TSA, and SLC8A1 (Abcam, Cat# ab2869) visualized with Opal 594 TSA. Slides were counterstained with DAPI for nuclei visualization for 10 min and coverslipped using the antifade mountant. All markers stained with multiplex immunofluorescence were reviewed by a pathologist.

### Single cell analysis

Single-cell transcriptome data (GSE162025) from 10 NPC tissues with 82,622 cells was analyzed using Seurat to deal with the expression matrix. By setting filtering parameters, we filtered out cells with mitochondrial gene ratio>10% to exclude cells in abnormal state, and filtered out cells with gene number<500 or >3000 to exclude data with poor sequencing quality and non-single cells, and finally obtained 75710 cells. We used the Harmony package for data integration and batch removal. We performed unsupervised clustering using the default parameters in the Seurat package and then annotated these cell populations. We used the FindAllMarkers function in the Seurat package to calculate the differential genes between cell populations (log2FC=0.25 and P<0.05). The obtained differential genes were sorted to get the top ten highly expressed genes with significant differences for each cell population, and the expression matrix of these genes was obtained using the FetchData function, and plotted using the corrplot package.

### Image analysis

All immunofluorescence slides were scanned using the Pannoramic Digital Slide Scanner (3DHistech) and images visualized in CaseViewer2.4 (3DHistech). TMA core images were analyzed in HALO (Indica Labs). Area Quantification FL V2.1 module in Halo V3.0.311.314 analysis software was used to quantify the positive area and colocalization-positive area of the target region, respectively. Extreme values (<10th percentile; > 90th percentile) were excluded to minimize data variability.

## Results

### Differential expression and enrichment analysis

**Figure 1:**
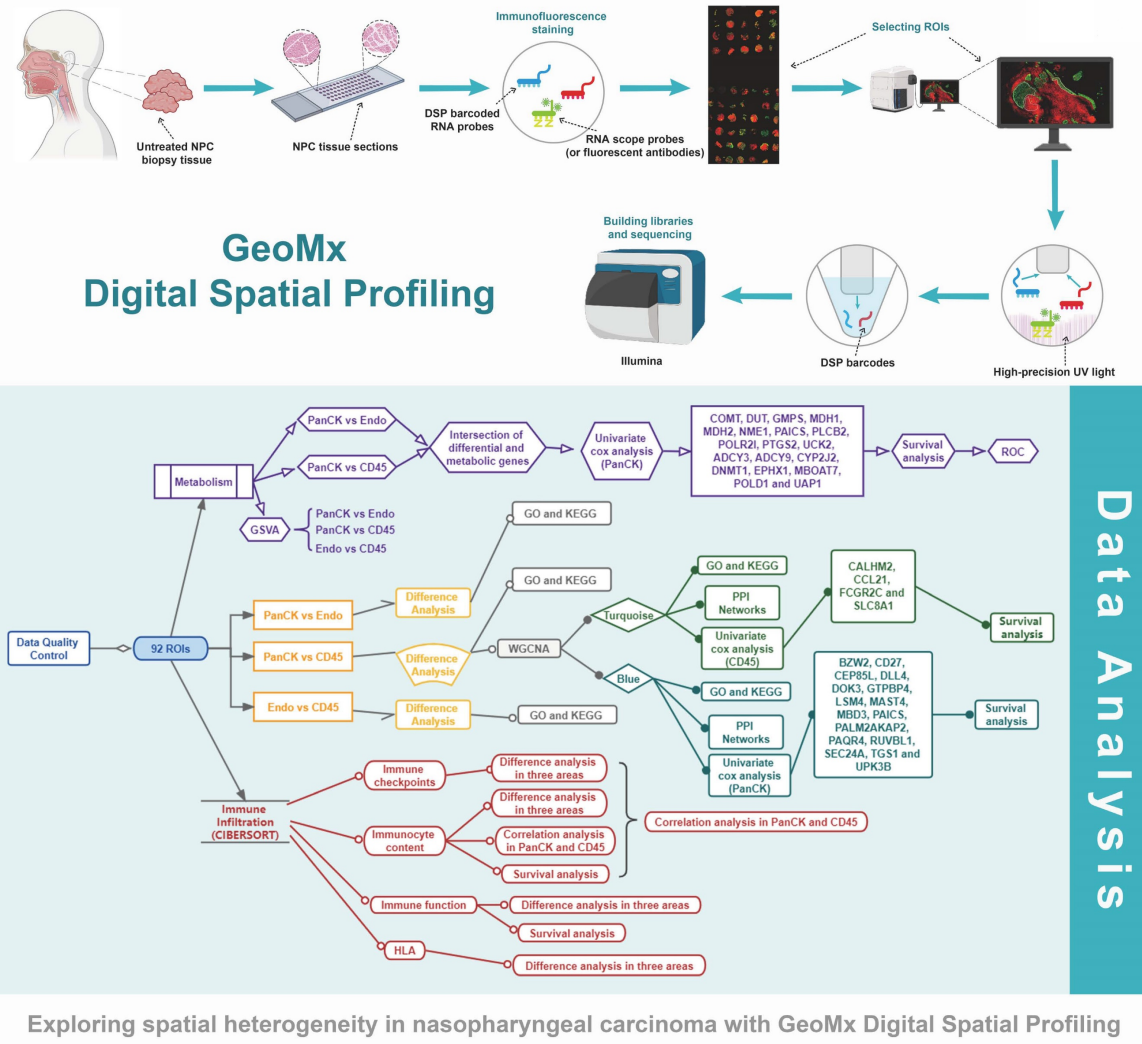
Flow chart of the study.

**Figure 2:**
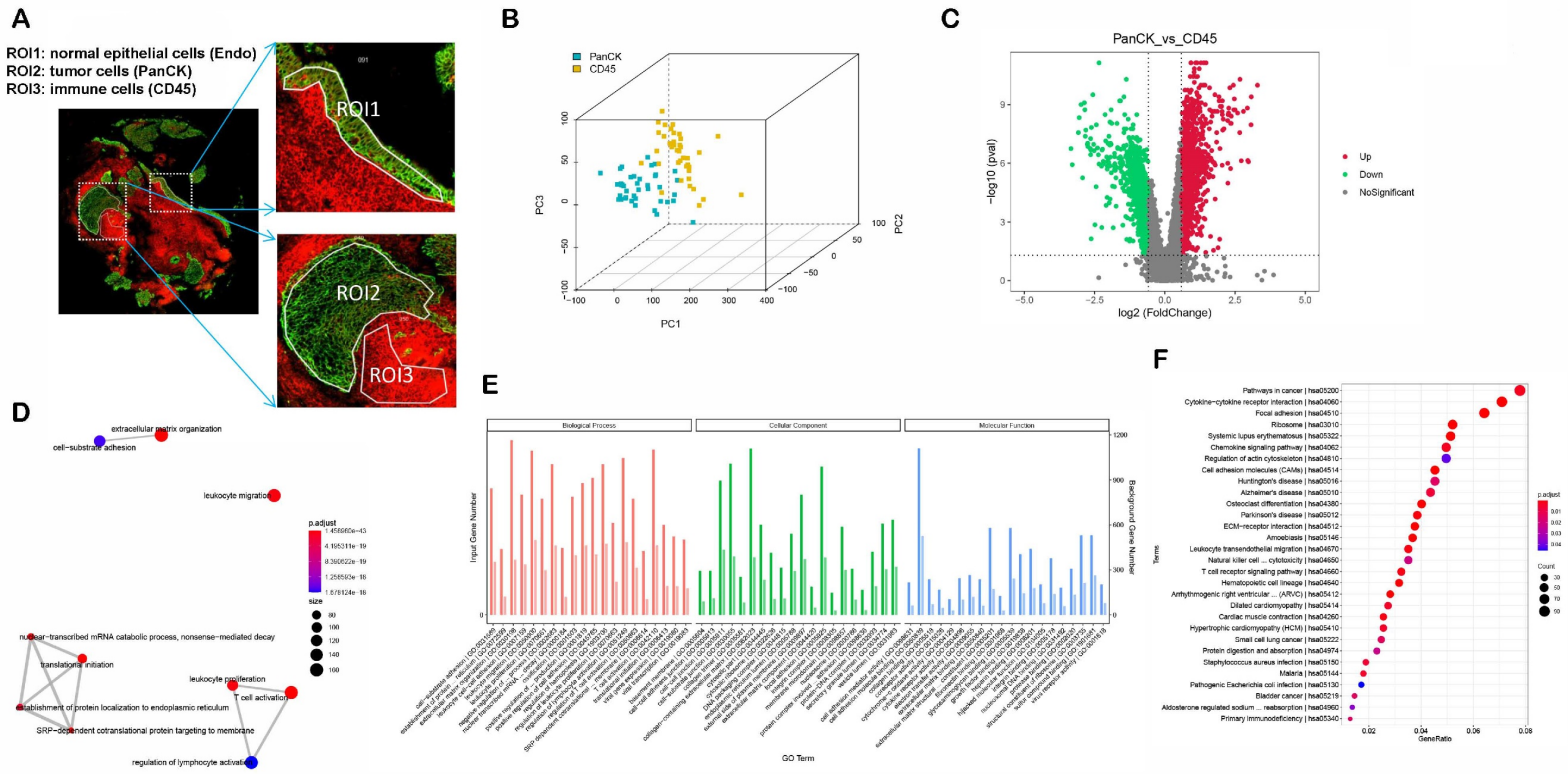
** Differential expression and enrichment analysis.** (A) Selecting ROIs: PanCK-expressing, CD45-expressing and Endo regions. (B) Principal Component Analysis. (C) Differential Analysis (PanCK-expressing vs CD45-expressing regions), red: upregulated genes; green: downregulated genes. (D-E) GO enrichment analysis of differentially expressed genes (PanCK-expressing vs. CD45-expressing regions). (F) KEGG enrichment analysis of differentially expressed genes (PanCK vs CD45).

The study protocol is shown in Figure 1. We included 41 patients with NPC using fluorescent anti-PanCK (an epithelial marker) and anti-CD45 (an immune marker) antibodies to label the ROIs. We distinguished normal epithelial cells from tumor cells based on pathologic morphology in the PanCK-expressing ROIs. Finally, a total of 40 tumor cell (PanCK-expressing) ROIs, 39 immune cell (CD45-expressing) and 13 normal epithelial cell (Endo) ROIs were labeled (Fig. 2A and Supplementary Table 1). The data from the validation cohort used for multiplex immunofluorescence are described in Supplementary Table 2. The results of principal component analysis are shown in Figure 2B, Figure s1. As shown in Figure 2C, differential gene expression across regions was counted from 18675 genes: 2940 (PanCK- vs CD45-expressing regions), 3660 (Endo vs CD45-expressing regions), and 2514 (PanCK-expressing vs Endo regions). These were determined by differential analysis with a fold change > 1.5 and p-values <0.05 (Fig. 2C, Fig. s1B and Fig.s1G). We performed GO and KEGG enrichment analysis of these differential genes. A total of 2940 differentially expressed genes (DEGs) between PanCK- vs CD45-expressing regions included those coding for products mainly localized to the basement membrane, cell-cell adherens junctions, and cell-cell junctions. These performed various functions including cell adhesion mediator activity, cell-adhesion molecule binding, and collagen-binding. They regulated pathways including cell adhesion molecules, cytokine-cytokine receptor interaction, and leukocyte transendothelial migration. They participated in biological processes including leukocyte cell-cell adhesion, leukocyte migration, and leukocyte proliferation (Fig. 2D, E and F). A total of 3660 DEGs in Endo vs CD45-expressing regions coded products mainly localized to the apical portion of the cell, apical plasma membrane, and axoneme. These gene products are involved in actin-binding, cell adhesion mediator activity, and cell adhesion molecule binding. They regulate pathways including ECM-receptor interaction, focal adhesion, and chemokine signaling pathway. They participate in biological processes including cell-substrate adhesion, extracellular matrix organization, and hemostasis (Fig. s1C, D and E). A total of 2514 DEGs in PanCK-expressing vs. Endo regions included those coding for products mainly localized to the axoneme, apical part of cell, and apical plasma membrane. These perform functions including ATP-dependent microtubule motor activity, cell adhesion molecule binding and DNA replication origin binding. They regulate pathways of the cell cycle, p53 signaling, apoptosis, and DNA replication. They participated in biological processes including cilium assembly, altered DNA conformation, and axoneme assembly (Fig. s1H, I and G).

### Analysis of regional core genes using WGCNA and PPI networks

**Figure 3:**
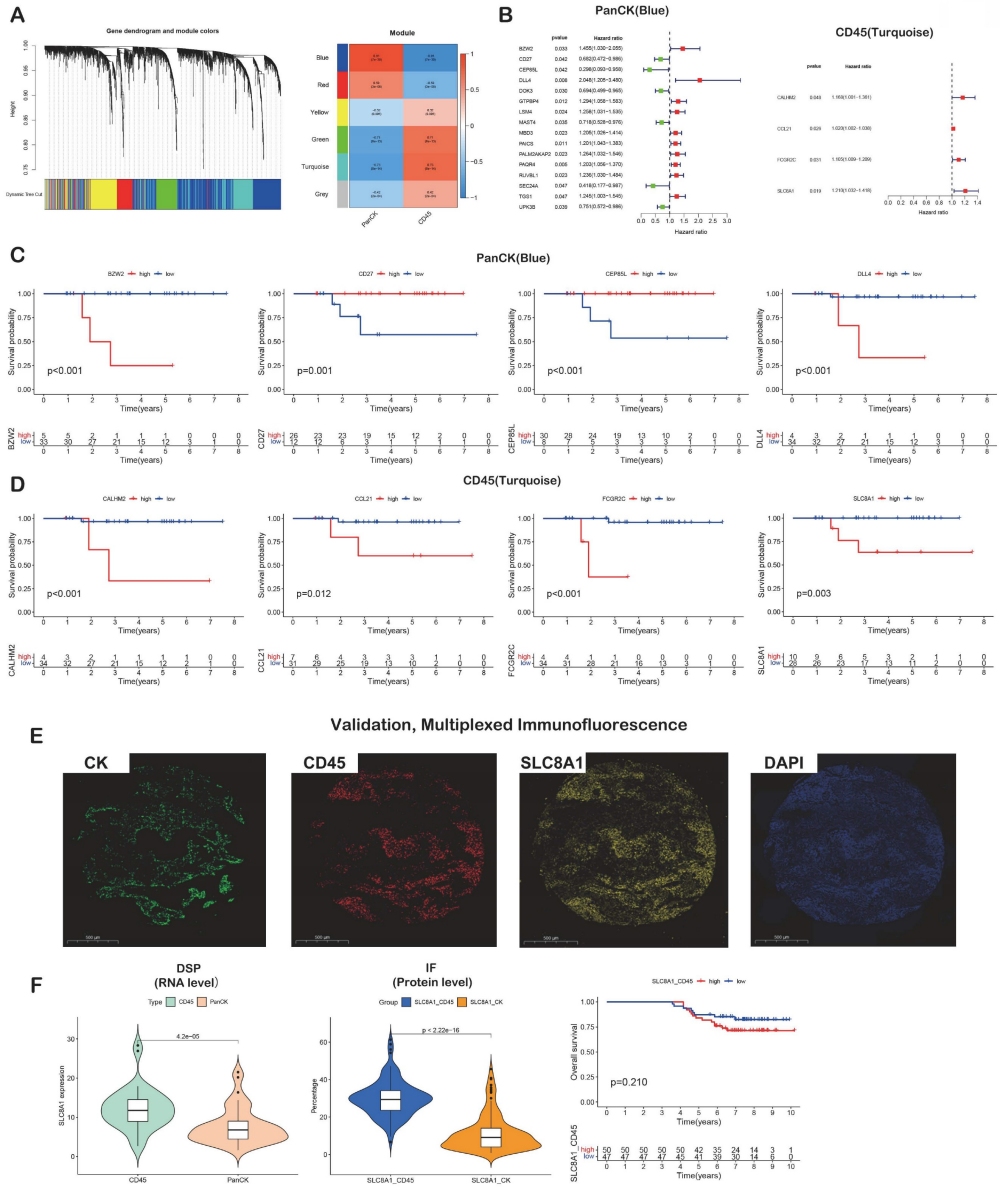
** Prognostic gene screening.** (A) WGCNA. (B) Genes in blue and turquoise modules were used for univariate Cox analysis in PanCK- and CD45-expressing regions, respectively. (C) Genes in blue module were used for survival analysis in PanCK-expressing regions. (D) Genes in turquoise modules were used for survival analysis in CD45-expressing regions. (E) Microphotographs of representative examples of staining in the multiplex immunofluorescence from the same patient core for each marker. (F) Differential RNA (left) and protein (middle) expression of SLC8A1 in CK and CD45 regions. The patients with higher SLC8A1 protein level in CD45 regions tends to have worse survival (right).

A total of 2940 DEGs between matched 38 PanCK- and 38 CD45-expressing regions were subjected to WGCNA. After merging dynamic modules by hierarchical clustering, a total of 6 modules were obtained. The blue module genes were significantly positively correlated with PanCK expression (r=0.91 and P=7e-30), while the turquoise module was significantly positively correlated with CD45 expression (r=0.73 and P= 8e-14) (Fig. 3A). A total of 1010 genes in the blue module coded for products mainly localized in cell-cell junctions, cell-substrate junctions, and focal adhesions. These genes play a role in molecular functions such as adhesion binding, cell adhesion mediator activity, and virus receptor activity. They are involved in biological processes such as epidermis development, cell junction assembly, and skin development (Fig. s2A). A total of 569 genes in the turquoise module mainly coded for products localized external to the plasma membranes, immunological synapses, and secretory granule membrane. These genes mediate C-C chemokine binding, GTPase regulator activity, and immune receptor activity. They are involved in biological processes such as T cell activation, lymphocyte proliferation, and lymphocyte differentiation (Fig. s2A). The genes of the blue module are mainly enriched in pathways involved in arrhythmogenic right ventricular cardiomyopathy, cellular adhesion, and ECM-receptor interaction. The genes of the turquoise module were mainly enriched in pathways such as chemokine signaling, natural killer cell-mediated cytotoxicity, and T cell receptor signaling (Fig. s2B). The blue modular genes including *CDK1, CCNB1, MCM2, TOP2A, CDC6, DTL, TPX2, UBE2C, KIF2C, RFC4, TYMS, MCM4, NUSAP1, MCM7, CENPF, FOXM1, RRM1, PCNA, MRPL13 and MRPL12* as the core genes were used to construct the PPI network. Turquoise modular genes including *CD4, CD8A, CD3E, CD247, ZAP70, LCK, CD3G, CD3D, FYN, VAV1, LCP2, CD28, ITK, PTPRC, LAT, CD2, CD80, PRKCQ, IL2RB and CTLA4* as the core genes were used to construct the PPI network (Fig. s2C). Enriched genes in the green module related to immune-related functions were not obvious (Fig. s2A, B and C).

### Analysis of prognostic gene

A total of 1010 genes in the blue module involving PanCK-expressing regions were screened for 16 prognosis-related genes via univariate Cox analysis (P <0.05). In PanCK-expressing regions, the genes *CD27*, *CEP85L*, *DOK3*, *MAST4*, *SEC24A* and *UPK3B* appeared to serve as protection factors. Conversely, the genes *BZW2*, *DLL4*, *GTPBP4*, *LSM4*, *MBD3*, *PAICS*, *PALM2AKAP2*, *PAQR4*, *RUVBL1* and *TGS1* represent risk factors; the higher the expression of these genes, the higher the risk of death in patients with NPC (Fig. 3B). Survival analysis showed that the higher expression of *CD27* (P =0.001), *CEP85L* (P < 0.001), *DOK3* (P <0.001), *MAST4* (P<0.001), *SEC24A* (P <0.001), and *UPK3B* (P <0.001) in PanCK-expressing regions of NPC patients correlated with better prognosis. In contrast, the expression of *BZW2* (P <0.001), *DLL4* (P <0.001), *GTPBP4* (P <0.001), *LSM4* (P <0.001), *MBD3* (P <0.001), *PAICS* (P <0.001), *PALM2AKAP2* (P =0.001), *PAQR4* (P <0.001), *RUVBL1* (P =0.006) and *TGS1* (P =0.124), correlated positively with worse prognosis in patients with NPC (Fig. 3C and Fig. s3). A total of 569 genes in the turquoise module involving CD45-expressing regions were screened for 4 prognosis-related genes via univariate Cox analysis (P <0.05). Higher expression of *CALHM2*, *CCL21*, *FCGR2C*, and *SLC8A1* was positively associated with poor survival in patients with NPC (Fig. 3B). Survival analysis showed that higher expression of *CALHM2* (P <0.001), *CCL21* (P =0.012), *FCGR2C* (P <0.001), and *SLC8A1* (P =0.003) was associated with poorer prognosis in patients with NPC (Fig. 3D). Due to the limitations of multi-color immunofluorescence techniques, we performed immunostaining including panCK, CD45 and SLC8A1. We selected SLC8A1 with the most obvious prognostic effect in the CD45 enriched region for verification (Fig. 3E). Both RNA level and protein level in CD45-expressing regions SLC8A1 was higher than that in PanCK-expressing regions (Fig. 3F). In CD45-expressing regions, the higher the expression of SLC8A1 (P = 0.210), the worse was the prognosis of NPC. Although statistically not significant, the trend was consistent with the transcriptional level (Fig. 3D).

### Immune infiltration of tumor tissue

**Figure 4:**
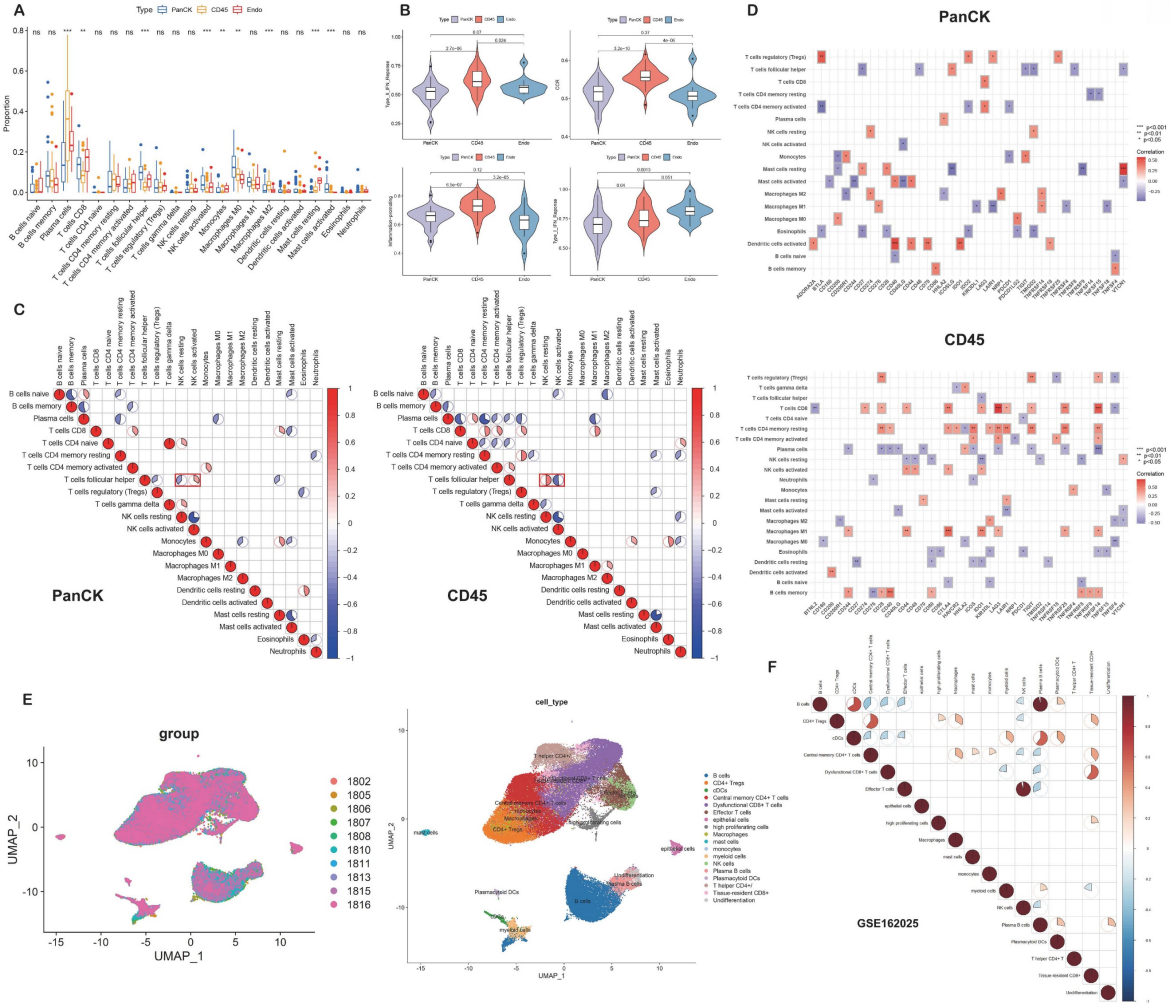
** Immune infiltration.** (A) Immune cell levels differed between the three groups. (B) Immune functions differed in three groups. (C) Correlation analysis of immune cells in PanCK- and CD45-expressing regions. (D) Correlation analysis of immune cells and immune checkpoints in PanCK- and CD45-expressing regions. (E) A UMAP plot of 75710 cells were divided into 18 clusters. Each dot represents a single cell, colored according to patients (left) and cell clusters (right). (F) Correlation analysis of immune cells in NPC (GSE162025).

**Figure 5:**
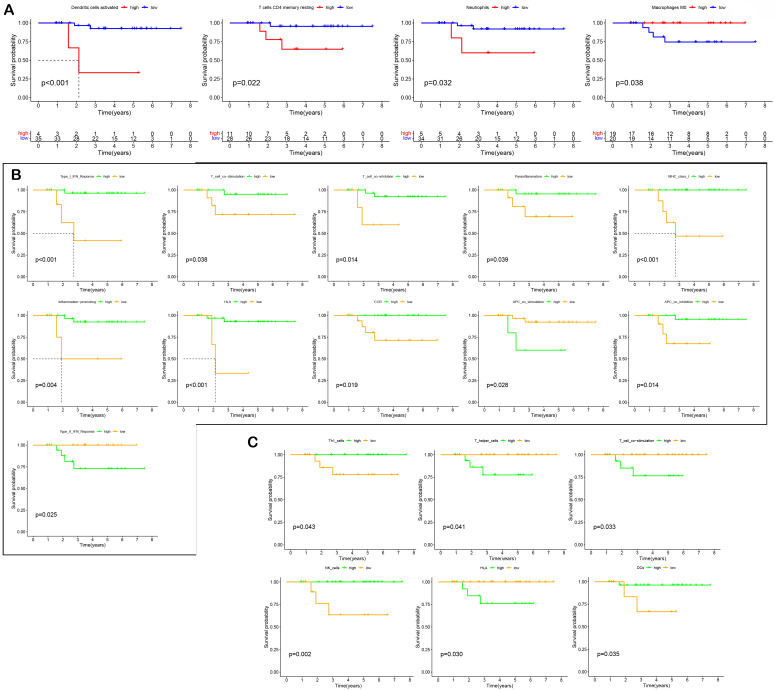
** Survival analysis.** (A) Effect of immune cell levels on survival in PanCK-expressing regions. (B) Effect of immune functions on survival in PanCK-expressing regions. (C) Effect of immune functions on survival in CD45-expressing regions.

The relative levels of 22 immune cells in 90 ROIs (39 PanCK-expressing, 38 CD45-expressing, and 13 Endo) are shown in Figure 4A and Supplementary Figure s4A. The number of plasma cells, CD8^+^ T cells, follicular helper T cells, activated NK cells, monocytes, M0 macrophages, M2 macrophages, resting mast cells, and activated mast cells differed among the three groups (P <0.05, 39 PanCK-expressing, 38 CD45-expressing and 13 Endo). The relative levels of plasma cells were the highest in CD45-expressing regions, while the number of CD8^+^ T, follicular helper T cells, and activated NK cells were the highest in PanCK-expressing regions. M0 macrophages were the highest in PanCK-expressing regions, while M2 macrophages were the highest in CD45-expressing regions (Fig. 4A). Differences in immune function of the three regions are displayed in Figure 4B and Supplementary Figure s4B. As shown in Figure 4C, the correlation of each type of immune cell differed between regions. In PanCK-expressing regions, follicular helper T cells were negatively correlated with resting NK cells (P <0.05) and positively correlated with activated NK cells (P <0.05). Conversely, in CD45-expressing regions, follicular helper T cells were positively correlated with resting NK cells (P <0.05) and negatively correlated with activated NK cells (P <0.05). In PanCK-expressing regions, Tregs were positively correlated with BTLA (P <0.01), resting mast cells with *VTCN1* (P <0.001), and activated dendritic cells with *CD40* (P <0.001) and *IDO1* (P <0.01). Conversely, negative correlations were found between CD4 memory activated T cells and *BTLA* (P <0.01), activated mast cells and *CD244* (P <0.01), and *CD40LG* (P <0.01), macrophages M1 and *LAIR1* (P <0.01) (Fig. 4D). In CD45-expressing regions, positive correlations were found between CD8^+^ T cells and *LAG3* (P <0.001) and *TNFSF14* (P <0.001), macrophages M1 and *CTLA4* (P <0.001), and B memory cells and *CD40* (P <0.001). Negative correlations were found between CD8^+^ T cells and *BTNL2* (P <0.01), plasma cells with *TNFSF14* (P <0.001), resting NK cells with *IDO1* (P <0.01), macrophages M0 and *TNFSF4* (P <0.01), resting dendritic cells and *CD27* (P <0.01), and B memory cells and *CD276* (P <0.01). We performed cluster annotation of 75710 cells from 10 NPC tissues and obtained a total of 18 cell populations (Fig. 4E). In the DSP dataset, B cells were positively correlated with plasma cell content and negatively correlated with most T cells and NK cells in NPC. This phenomenon was verified in the single-cell dataset (Fig. 4F). In PanCK-expressing regions, a worse prognosis of NPC was associated with higher levels of activated dendritic cells (p<0.001), CD4 memory resting T cells (p =0.022) and neutrophils (p =0.032). Conversely, higher levels of M0 macrophages correlated with a better prognosis of NPC (p = 0.038) (Fig. 5A). The survival analysis of immune function in PanCK- and CD45-expressing regions is shown in Figures 5B and 5C.

### HLA and immune checkpoints

**Figure 6:**
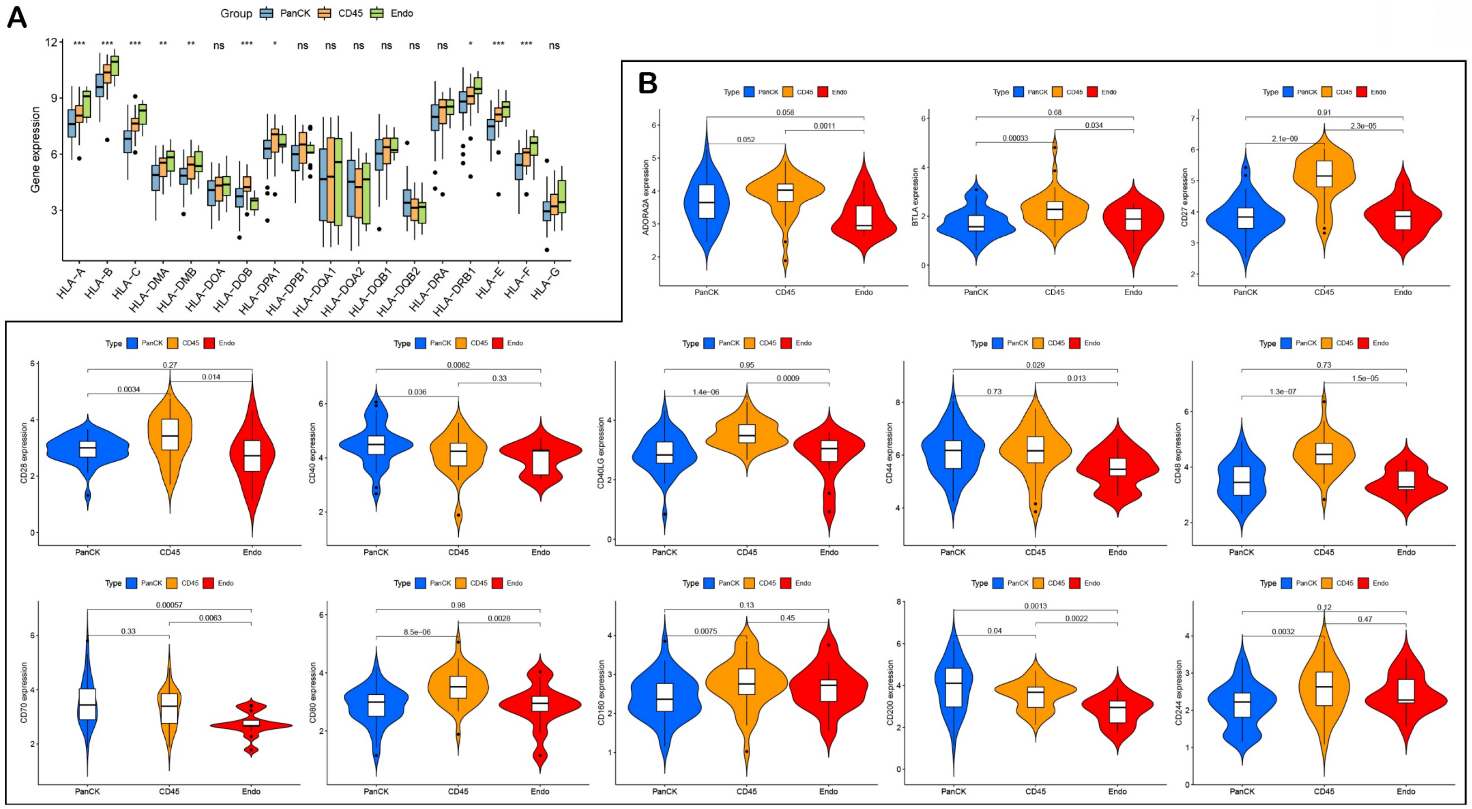
** HLA and immune checkpoints.** (A) Differences in HLA-related genes of the three regions. (B) Differences in immune checkpoint-related genes between the three regions.

The expression of HLA genes differed in the three regions (P <0.05) (Fig. 6A). The expression of immune checkpoint-related genes differed in the different regions. The expression of *CD200* (P =0.04), *CD40* (P =0.036) and *ICOSLG* (P =1.4e-05) was higher in PanCK-expressing regions than in CD45. The expression of *CD244* (P =0.0032), *CD160* (P =0.0075), *CD80* (P =8.5e-06), *CD48* (P =1.3e-07), *CD40LG* (P =1.4e-06), *CD28* (P =0.0034), *CD27* (P =2.1e-09), *BTLA* (P =0.00033), *ICOS* (P =0.00075), *HAVCR2* (P =0.0044), *CTLA4* (P =2e-06), *NRP1* (P =1.4e-09), *LAIR1* (P =1e-05), *LAG3* (P =0.0054), *PDCD1LG2* (P =0.0016), *TNFRSF8* (P =0.017), *TNFRSF4* (P =0.021), *TMIGD2* (P =0.019), *TIGIT* (P =8.2e-05), and *TNFSF18* (P =0.0033) was higher in CD45-expressing regions than in PanCK-expressing regions. In Endo regions, the expression of *CD200* (P =0.0013), *CD70* (P =0.00057), *CD44* (P =0.029), *CD40* (P =0.0062), *CD276* (P =0.0043), *ICOSLG* (P =1.1e-05), *TNFRSF9* (P =0.0016), and *TNFSF4* (P =0.0038) was lower than in PanCK-expressing regions, while the expression of *NRP1* (P =0.046), *IDO1* (P =0.0056), and *VTCN1* (P =0.0025) was higher than in PanCK-expressing regions (Fig. s5 and Fig. 6B).

### Metabolism-related genes

**Figure 7:**
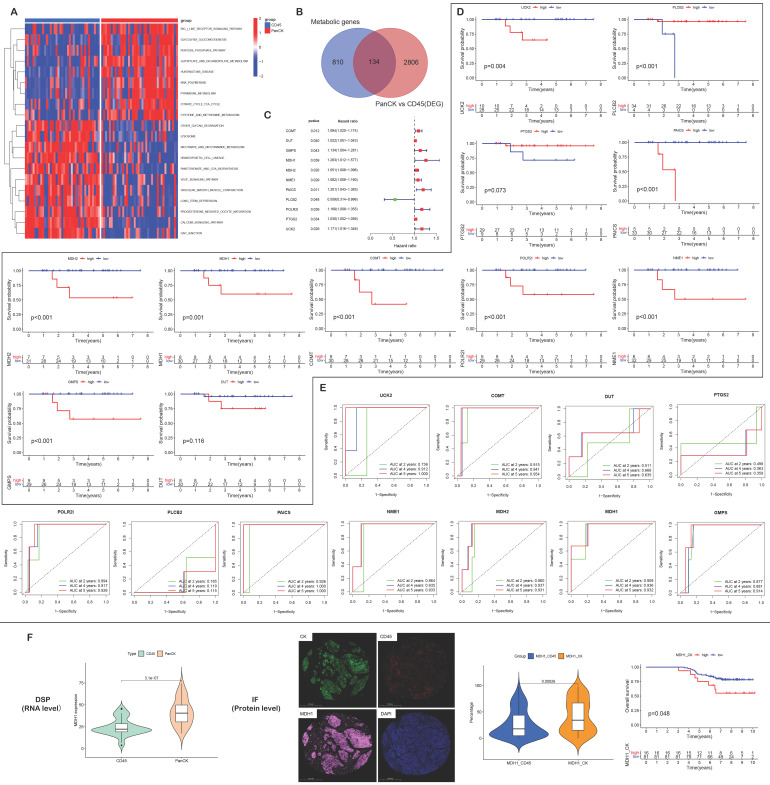
** Metabolism-related genes.** (A) GSVA enrichment analysis of PanCK- and CD45-expressing regions. (B) 944 metabolic genes, 2940 differentially expressed genes (PanCK- vs. CD45-expressing regions) and 134 intersecting genes. (C) Univariate Cox analysis of 134 intersecting genes in PanCK-expressing regions. (D) Survival analysis of prognostic metabolic genes in PanCK-expressing regions. (E) ROC curves: predicting survival rates of patients with NPC. (F) Differential RNA (left) and protein (middle) expression of MDH1 in Pan-CK and CD45 regions. The patients with higher MDH1 protein level in Pan-CK regions tends to have worse survival (right).

In PanCK-expressing regions, metabolic genes (Figure 7A) were mainly enriched in activities pertaining to glycoisomerization, the pentose phosphate pathway, glyoxylate and dicarboxylic acid metabolism, ribonucleic acid polymerase, pyrimidine metabolism, the citric acid cycle, and cysteine and methionine metabolism (P <0.05). In CD45-expressing regions, metabolic genes were mainly enriched in activities pertaining to lysosomes, nicotinate and nicotinamide metabolism, pantothenate and coenzyme biosynthesis, the vascular endothelial growth factor signaling pathway, vascular smooth muscle contraction, progesterone-mediated oocyte maturation, and the calcium signaling pathway (P <0.05). As shown in Supplementary Figure s6A, metabolic pathways showed that in PanCK-expressing regions, metabolic genes were mainly enriched in primary immunodeficiency, Fcγ-mediated phagocytosis, regulation of actin cytoskeleton, pathways in cancer, the T cell receptor signaling pathway, P53 signaling, the one-carbon pool by folate pathway, DNA replication, purine metabolism, pyrimidine metabolism, glyoxylate and dicarboxylate metabolism, and cysteine and methionine metabolism (P <0.05). In Endo regions, metabolic genes were mainly enriched in histidine metabolism, peroxisome, metabolism of xenobiotics by cytochrome p450, drug metabolism by cytochrome p450, sphingolipid metabolism, and both amino sugar and nucleotide sugar metabolism (P <0.05).

A total of 134 genes were obtained by intersecting 944 metabolic genes with 2940 differential genes (PanCK-expressing vs CD45-expressing regions) (Fig. 7B). Univariate Cox regression analysis of 134 genes in PanCK*-*expressing regions revealed 11 prognosis-related metabolic genes. The expression of *COMT* (P = 0.012), DUT (P =0.040), GMPS (P =0.043), *MDH1* (P = 0.039), *MDH2* (P = 0.020), NME1 (P =0.029), *PAICS* (P =0.011), POLR2I (P =0.039), *PTGS2* (P =0.034) and UCK2 (P =0.029) increased the risk of NPC, while *PLCB2* (P = 0.048) was a protective factor (Fig. 7C). In PanCK-expressing regions, the higher the expression of *COMT* (P <0.001), *GMPS* (P <0.001), *MDH1* (P =0.001), *MDH2* (P <0.001), *NME1* (P <0.001), *PAICS* (P <0.001), *POLR2I* (P <0.001), and *UCK2* (P =0.004), the worse was the prognosis of NPC. In contrast, a higher expression of *PLCB2* (P <0.001) was associated with a better prognosis for survival in patients with in NPC (Fig. 7D). The ROC curves predicting 2-, 4-, and 5-year survival of patients with NPC are shown in Figure 7E. Among the differentially expressed metabolic genes in panCK and CD45 regions, MDH1 which has the most obvious prognostic effect was selected for validation. The expression of *MDH1* (AUC = 0.908, 0.936 and 0.932) predicted 2-, 4-, and 5-year survival rates in patients with NPC with a high degree of accuracy. MDH1 expression is higher in PanCK than in CD45 (P < 0.001). Multiplexed immunofluorescence analysis revealed higher levels of MDH1 protein in PanCK than in CD45 (P < 0.001), and the higher the MDH1 protein level (P = 0.048), the worse was the prognosis of patients with NPC (Fig. 7F).

A total of 171 genes were obtained by intersecting 944 metabolic genes with 2514 differential genes (PanCK-expressing vs Endo regions) (Fig. s6B). Univariate Cox regression analysis of 171 genes in PanCK-expressing regions and 13 prognosis-related metabolic genes revealed that the expression of *ADCY3* (P = 0.027), *ADCY9* (P =0.028), *CYP2J2 (P = 0.038)*, *DNMT1* (P = 0.024), *EPHX1* (P =0.022), *MBOAT7* (P =0.018), *POLD1 (P = 0.016) and UAP1 (P = 0.041)* correlated positively with survival risk in patients with NPC (Fig. S6C). Notably, *DUT, NME1, UCK2*, *PAICS* and *PTGS2* were both screened out in two univariate Cox analyses. *DUT, NME1, UCK2, PAICS* and *PTGS2* were differentially expressed between regions (PanCK-expressing vs. CD45-expressing regions and PanCK-expressing vs Endo regions), and were also metabolism-related genes. The expression of these genes correlated with a higher risk of death in patients with NPC.Higher expression of *ADCY3* (P = 0.005), *ADCY9* (P =0.008), *CYP2J2 (*P = 0.001), *DNMT1* (P <0.001), *EPHX1* (P <0.001), *MBOAT7* (P <0.001), *POLD1* (P = 0.001) and *UAP1* (P < 0.001) was associated with worse prognosis in patients with NPC (Fig. S6D). The ROC curves for these NPC prognostic genes are presented in Supplementary Figure s6E.

## Discussion

Either bulk-RNA seq or microarray sequencing was used to explore biomarkers of NPC before GeoMx DSP technology emerged 12. However, these sequencing techniques only generated the average gene expression of all the components found in tumor tissue. The screened markers did not identify the region or cell expressing marker. This greatly limited the exploration of the NPC microenvironment.

Using DSP technology, we screened 20 prognostic gene markers located in tumor cell-enriched regions (*CEP85L*, *KRT5*, *MAST4*, *MYO1G*, *SLA*, *SMARCC2*, *UPK3B*, *ATF5*, *BEX3*, *BIK*, *CADM4*, *CDK2AP1*, *CLDN1*, *DLL4*, *IGFBP2*, *NFE2L3*, *PTGS2*, *SMC1A*, *SUSD4* and *VRK2*) and 4 prognostic markers in immune cell-enriched regions (*CALHM2*, *CCL21*, *FCGR2C* and *SLC8A1*). We also screened for metabolism-related genes that affect the prognosis of patients with NPC in tumor cell-enriched regions (*COMT, DUT, GMPS, MDH1, MDH2, NME1, PAICS, PLCB2, POLR2I, PTGS2, UCK2, ADCY3, ADCY9, CYP2J2, DNMT1, EPHX1, MBOAT7, POLD1 and UAP1*).

The gene MDH1 regulates autophagy in pancreatic ductal adenocarcinoma (PDAC) associated with PDAC cell survival 13. O-GlcNAcylation enhances MDH1 activity to promote proliferation of pancreatic ductal adenocarcinoma cells. MDH1 may be a prognostic marker of esophageal squamous cell carcinoma 14. The levels of MDH1 and MDH2 enzymes in the cytoplasm and mitochondria of non-small-cell lung cancer (NSCLC) cells have been shown to be elevated compared with normal cells, and MDH1 enzyme activity was significantly higher than that of MDH2. However, only MDH1 expression was associated with poor prognosis in patients diagnosed with NSCLC 15, 16. MDH1 is predicted to be a prognostic marker of lung squamous cell carcinoma (LUSC) based on multivariate Cox regression analysis 17. In addition, the frequency of MDH1 mutations in LUSC is 5% in the cBioPortal database 17. SLC8A1 downstream intergenic region ALK fusion was detected in patients with advanced lung adenocarcinoma 18. SLC8A1 is associated with survival of breast cancer and colorectal neoplasia 19, 20. Few studies have reported the roles of MDH1 and SLC8A1 in NPC or explored the impact of their spatial distribution on the prognosis of tumor patients. Therefore, we explored the effect of the spatial location of MDH1 and SLC8A1 on the prognosis of patients with NPC. At the transcriptional level, MDH1 expression is higher in PanCK expressing regions than in CD45 expressing regions, and the higher its expression, the worse the prognosis of NPC. The expression of SLC8A1 is higher in CD45 expressing regions than in PanCK expressing regions, and the higher its expression, the worse the prognosis. The protein levels of MDH1 are higher in PanCK expressing regions than in CD45 expressing regions, and the higher level worsened the prognosis. The protein level of SLC8A1 is higher in CD45 expressing regions than in PanCK expressing regions.

The predominantly infiltrating lymphocytes in the NPC microenvironment are T (CD8^+^ T, CD4^+^ T-helper, naïve T-cells, cytotoxic T-cells, exhausted T-cells, and Tregs) and B cells 7, 21. In previous transcriptome sequencing studies, both CD4^+^ and CD8^+^ T cell clusters in NPC were highly activated and depleted and co-expressed effector markers such as IL-2, GZMB, INFG, NKG7, GNLY, and GZMK. They also expressed depletion markers such as PDCD1, HAVCR2, LAG3, TIGIT, and CTLA4 22, 23. In our study, the levels of CD8^+^ T, follicular helper T cells, activated NK cells, and M0 macrophages were higher in tumor cell-enriched regions than in immune cell-enriched regions. In contrast, plasma cells and M2 macrophages were more abundant in immune cell-enriched regions, suggesting that cellular immunity was predominantly exerted in tumor cell-enriched regions, while humoral immunity was mainly observed in the peritumor area. Interestingly, in tumor cell-enriched regions, follicular helper T cells were negatively correlated with resting NK cells and positively correlated with activated NK cells. In immune cell-enriched regions, the opposite was true, highlighting the need to use single-cell sequencing to further explore the interactions between these two cell types. In tumor cell-enriched regions, PDCD1 expression was negatively correlated with the levels of monocytes and CD4^+^ memory-activated T cells. The expression of LAG3 positively correlated with the levels of CD4^+^ memory-activated T cells and CD8 T cells. The expression of TIGIT positively correlated with monocyte levels and negatively correlated with follicular helper T cell levels. In immune cell-enriched regions, the PDCD1 expression negatively correlated with the number of eosinophils and CD4^+^ naïve T cells. The expression of HAVCR2 negatively correlated with the number of gamma/delta T cells and positively correlated with the levels of CD4^+^ memory resting T cells. The expression of LAG3 positively correlated with the levels of CD4^+^ memory activated T cells, CD4^+^ memory resting T cells, M1 macrophages, and CD8^+^ T cells, but negatively correlated with the number of plasma cells. The expression of TIGIT was positively related with the levels of Tregs, CD8^+^ T cells, and CD4^+^ memory resting T cells, while negatively correlated with the number of plasma cells. The expression of CTLA4 positively correlated with the levels of CD8^+^ T cells, CD4^+^ memory resting T cells, activated NK cells, and M1 macrophages, while negatively correlated with the number of naive B cells. We therefore analyzed the relationship between these immune cells and depletion markers in locations that were complementary to previous studies. However, the specific mechanisms of interactions between cells at various spatial locations need to be further explored.

Limitations of this study include the small sample size, which restricts the correlation between the tumor immune characteristics, treatment, and outcomes. Even though our study is small the strength is the paired samples. Another limitation is that the biomarkers we screened have not yet been validated in functional experiments, and the roles of these biomarkers still need to be further elucidated through in vivo and in vitro experiments in the future.

In conclusion, we used DSP technology to explore the heterogeneity of the NPC microenvironment and its regional and metabolic prognostic markers and characterized the immune infiltration. The screened prognostic biomarkers may provide a theoretical basis for clinical diagnosis, targeted therapy, and new drug development.

## Supplementary Material

Supplementary figures and tables.
