# Supplementary material for: Exploration of spatial heterogeneity of tumor microenvironment in nasopharyngeal carcinoma via transcriptional digital spatial profiling
Source: Int J Biol Sci. 2023 Apr 17;19(7):2256–69. doi: 10.7150/ijbs.74653 (PMC10158028; doi:10.7150/ijbs.74653)
Supplement: Supplementary file 1 — Supplementary figures and tables. [file ijbsv19p2256s1.pdf]

Figure legends

Figure s1. Differential expression and enrichment analysis. (A) Principal Component Analysis (Endo vs CD45). (B) Difference Analysis (Endo vs CD45), red: upregulated genes; green: downregulated genes. (C) GO enrichment analysis of differential genes (Endo vs CD45). (D) KEGG enrichment analysis of differential genes (Endo vs CD45). (E) Biological process-related networks (Endo vs CD45). (F) Principal Component Analysis (PanCK vs Endo). (G) Difference Analysis (PanCK vs Endo), red: upregulated genes; green: downregulated genes. (H) GO enrichment analysis of differential genes (PanCK vs Endo). (I) KEGG enrichment analysis of differential genes (PanCK vs Endo). (J) Biological process-related networks (PanCK vs Endo).

Figure s2. Functional enrichment analysis and PPI network construction. (A) Genes in blue, turquoise and green modules were used for GO enrichment analysis. (B) Genes in blue, turquoise and green modules were used for KEGG enrichment analysis. (C) Genes in blue, turquoise and green modules were used to build the PPI network.

Figure s3. Genes in blue modules were used for survival analysis in PanCK.

Figure s4. Immune infiltration. (A) Relative levels of immune cells in each ROI. (B) Immune functions differed in three groups.

Figure s5. Differences between immune checkpoint-related genes in the three regions.

Figure s6. Metabolism-related genes. (A) GSVA enrichment analysis: PanCK vs. Endo and CD45 vs. Endo. (B) 944 metabolic genes, 2514 differential genes (PanCK vs. Endo) and 171 intersecting genes. (C) Univariate Cox regression analysis of 171 intersecting genes in PanCK. (D) Survival analysis of prognostic metabolic genes in PanCK. (E) ROC curves: predicting 2-, 4-, and 5-year survival rates of patients with NPC.

Fig. s1

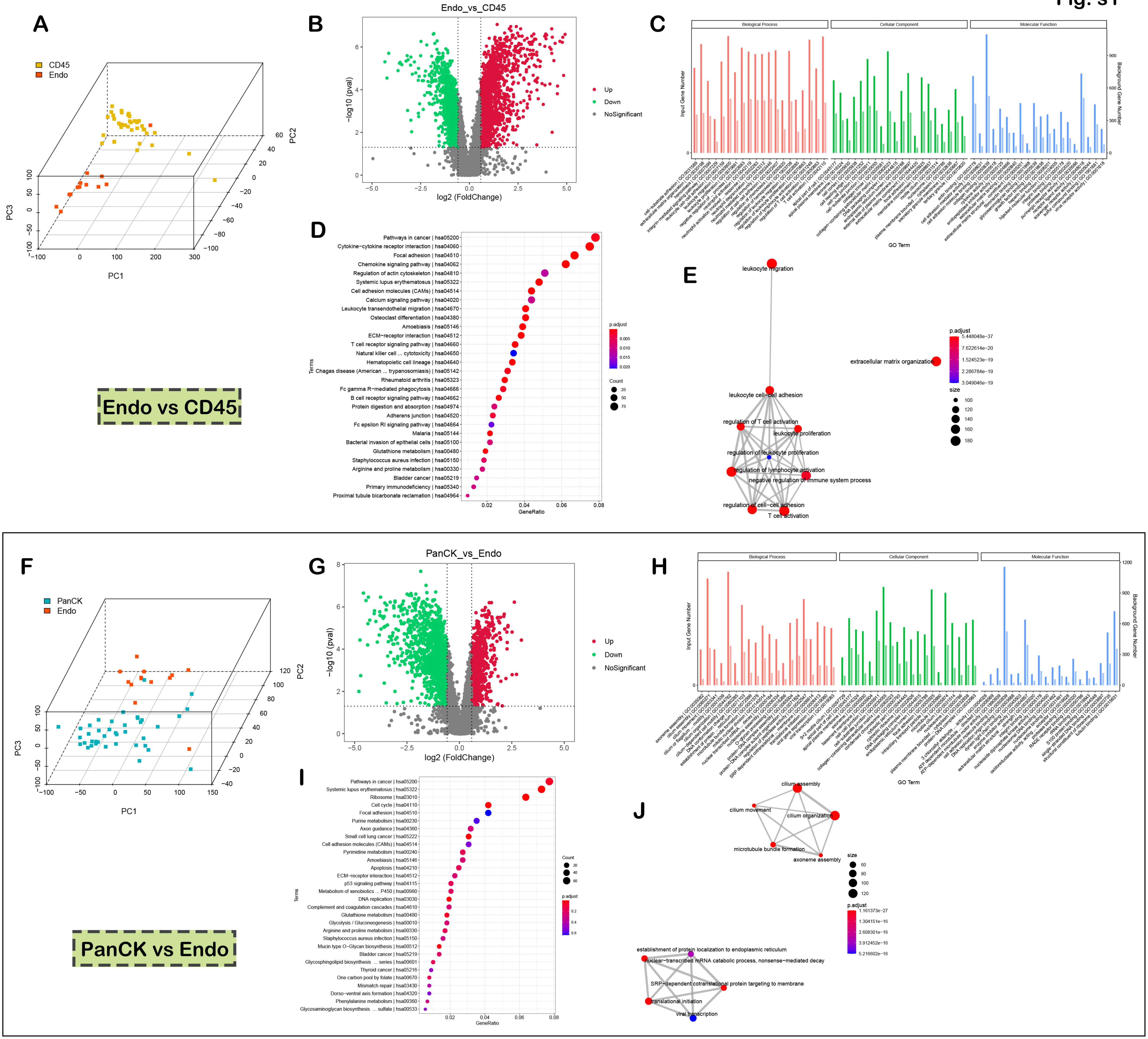

A
CD45
Endo
PC1
PC2
PC3
Endo vs CD45
B
Endo_vs_CD45
−log10 (pval)
log2 (FoldChange)
Up
Down
NoSignificant
C
Biological Process
Cellular Component
Molecular Function
Input Gene Number
Background Gene Number
GO Term
D
Pathways in cancer | hsa05200
Cytokine−cytokine receptor interaction | hsa04060
Focal adhesion | hsa04510
Chemokine signaling pathway | hsa04062
Regulation of actin cytoskeleton | hsa04810
Systemic lupus erythematosus | hsa05322
Cell adhesion molecules (CAMs) | hsa04514
Calcium signaling pathway | hsa04020
Leukocyte transendothelial migration | hsa04670
Osteoclast differentiation | hsa04380
Amoebiasis | hsa05146
ECM−receptor interaction | hsa04512
T cell receptor signaling pathway | hsa04660
Natural killer cell ... cytotoxicity | hsa04650
Hematopoietic cell lineage | hsa04640
Chagas disease (American ... trypanosomiasis) | hsa05142
Rheumatoid arthritis | hsa05323
Fc gamma R−mediated phagocytosis | hsa04666
B cell receptor signaling pathway | hsa04662
Protein digestion and absorption | hsa04974
Adherens junction | hsa04520
Fc epsilon RI signaling pathway | hsa04664
Malaria | hsa05144
Bacterial invasion of epithelial cells | hsa05100
Glutathione metabolism | hsa00480
Staphylococcus aureus infection | hsa05150
Arginine and proline metabolism | hsa00330
Bladder cancer | hsa05219
Primary immunodeficiency | hsa05340
Proximal tubule bicarbonate reclamation | hsa04964
Terms
GeneRatio
p.adjust
Count
E
leukocyte migration
extracellular matrix organization
leukocyte cell−cell adhesion
regulation of T cell activation
leukocyte proliferation
regulation of leukocyte proliferation
regulation of lymphocyte activation
negative regulation of immune system process
regulation of cell−cell adhesion
T cell activation
p.adjust
size
F
PanCK
Endo
PC1
PC2
PC3
PanCK vs Endo
G
PanCK_vs_Endo
−log10 (pval)
log2 (FoldChange)
Up
Down
NoSignificant
H
Biological Process
Cellular Component
Molecular Function
Input Gene Number
Background Gene Number
GO Term
I
Pathways in cancer | hsa05200
Systemic lupus erythematosus | hsa05322
Ribosome | hsa03010
Cell cycle | hsa04110
Focal adhesion | hsa04510
Purine metabolism | hsa00230
Axon guidance | hsa04360
Small cell lung cancer | hsa05222
Cell adhesion molecules (CAMs) | hsa04514
Pyrimidine metabolism | hsa00240
Amoebiasis | hsa05146
Apoptosis | hsa04210
ECM−receptor interaction | hsa04512
p53 signaling pathway | hsa04115
Metabolism of xenobiotics ... P450 | hsa00980
DNA replication | hsa03030
Complement and coagulation cascades | hsa04610
Glutathione metabolism | hsa00480
Glycolysis / Gluconeogenesis | hsa00010
Arginine and proline metabolism | hsa00330
Staphylococcus aureus infection | hsa05150
Mucin type O−Glycan biosynthesis | hsa00512
Bladder cancer | hsa05219
Glycosphingolipid biosynthesis ... series | hsa00601
Thyroid cancer | hsa05216
One carbon pool by folate | hsa00670
Mismatch repair | hsa03430
Dorso−ventral axis formation | hsa04320
Phenylalanine metabolism | hsa00360
Glycosaminoglycan biosynthesis ... sulfate | hsa00533
Terms
GeneRatio
Count
p.adjust
J
cilium assembly
cilium movement
cilium organization
microtubule bundle formation
axoneme assembly
size
p.adjust
establishment of protein localization to endoplasmic reticulum
nuclear−transcribed mRNA catabolic process, nonsense−mediated decay
SRP−dependent cotranslational protein targeting to membrane
translational initiation
viral transcription

Fig. s2

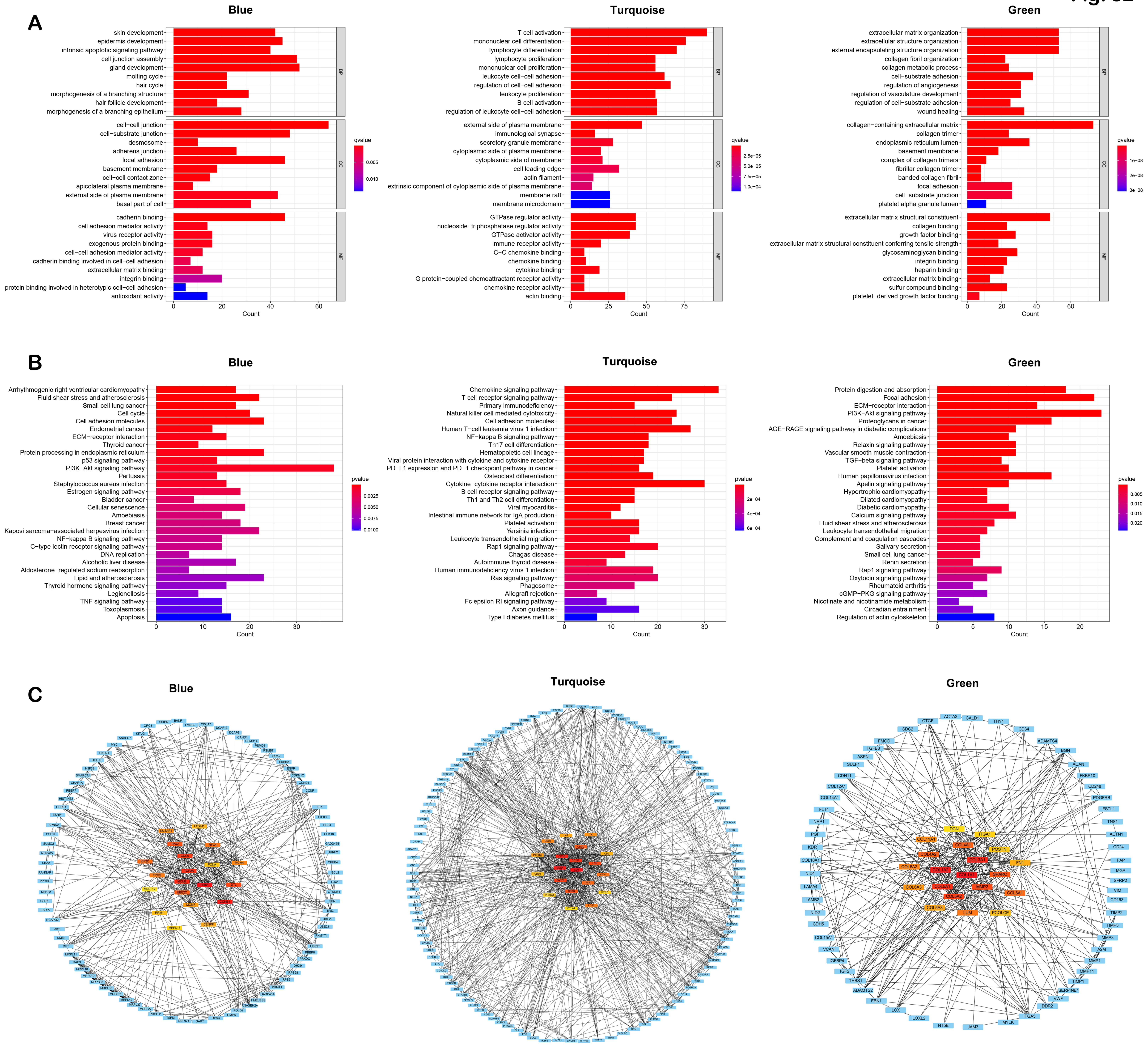
A
Blue
skin development
epidermis development
intrinsic apoptotic signaling pathway
cell junction assembly
gland development
molting cycle
hair cycle
morphogenesis of a branching structure
hair follicle development
morphogenesis of a branching epithelium
cell−cell junction
cell−substrate junction
desmosome
adherens junction
focal adhesion
basement membrane
cell−cell contact zone
apicolateral plasma membrane
external side of plasma membrane
basal part of cell
cadherin binding
cell adhesion mediator activity
virus receptor activity
exogenous protein binding
cell−cell adhesion mediator activity
cadherin binding involved in cell−cell adhesion
extracellular matrix binding
integrin binding
protein binding involved in heterotypic cell−cell adhesion
antioxidant activity
BP
CC
MF
qvalue
0.005
0.010
0 20 40 60
Count
Turquoise
T cell activation
mononuclear cell differentiation
lymphocyte differentiation
lymphocyte proliferation
mononuclear cell proliferation
leukocyte cell−cell adhesion
regulation of cell−cell adhesion
leukocyte proliferation
B cell activation
regulation of leukocyte cell−cell adhesion
external side of plasma membrane
immunological synapse
secretory granule membrane
cytoplasmic side of plasma membrane
cytoplasmic side of membrane
cell leading edge
actin filament
extrinsic component of cytoplasmic side of plasma membrane
membrane raft
membrane microdomain
GTPase regulator activity
nucleoside−triphosphatase regulator activity
GTPase activator activity
immune receptor activity
C−C chemokine binding
chemokine binding
cytokine binding
G protein−coupled chemoattractant receptor activity
chemokine receptor activity
actin binding
qvalue
2.5e−05
5.0e−05
7.5e−05
1.0e−04
0 25 50 75
Count
Green
extracellular matrix organization
extracellular structure organization
external encapsulating structure organization
collagen fibril organization
collagen metabolic process
cell−substrate adhesion
regulation of angiogenesis
regulation of vasculature development
regulation of cell−substrate adhesion
wound healing
collagen−containing extracellular matrix
collagen trimer
endoplasmic reticulum lumen
basement membrane
complex of collagen trimers
fibrillar collagen trimer
banded collagen fibril
focal adhesion
cell−substrate junction
platelet alpha granule lumen
extracellular matrix structural constituent
collagen binding
growth factor binding
extracellular matrix structural constituent conferring tensile strength
glycosaminoglycan binding
integrin binding
heparin binding
extracellular matrix binding
sulfur compound binding
platelet−derived growth factor binding
qvalue
1e−08
2e−08
3e−08
0 20 40 60
Count
B
Blue
Arrhythmogenic right ventricular cardiomyopathy
Fluid shear stress and atherosclerosis
Small cell lung cancer
Cell cycle
Cell adhesion molecules
Endometrial cancer
ECM−receptor interaction
Thyroid cancer
Protein processing in endoplasmic reticulum
p53 signaling pathway
PI3K−Akt signaling pathway
Pertussis
Staphylococcus aureus infection
Estrogen signaling pathway
Bladder cancer
Cellular senescence
Amoebiasis
Breast cancer
Kaposi sarcoma−associated herpesvirus infection
NF−kappa B signaling pathway
C−type lectin receptor signaling pathway
DNA replication
Alcoholic liver disease
Aldosterone−regulated sodium reabsorption
Lipid and atherosclerosis
Thyroid hormone signaling pathway
Legionellosis
TNF signaling pathway
Toxoplasmosis
Apoptosis
pvalue
0.0025
0.0050
0.0075
0.0100
0 10 20 30
Count
Turquoise
Chemokine signaling pathway
T cell receptor signaling pathway
Primary immunodeficiency
Natural killer cell mediated cytotoxicity
Cell adhesion molecules
Human T−cell leukemia virus 1 infection
NF−kappa B signaling pathway
Th17 cell differentiation
Hematopoietic cell lineage
Viral protein interaction with cytokine and cytokine receptor
PD−L1 expression and PD−1 checkpoint pathway in cancer
Osteoclast differentiation
Cytokine−cytokine receptor interaction
B cell receptor signaling pathway
Th1 and Th2 cell differentiation
Viral myocarditis
Intestinal immune network for IgA production
Platelet activation
Yersinia infection
Leukocyte transendothelial migration
Rap1 signaling pathway
Chagas disease
Autoimmune thyroid disease
Human immunodeficiency virus 1 infection
Ras signaling pathway
Phagosome
Allograft rejection
Fc epsilon RI signaling pathway
Axon guidance
Type I diabetes mellitus
pvalue
2e−04
4e−04
6e−04
0 10 20 30
Count
Green
Protein digestion and absorption
Focal adhesion
ECM−receptor interaction
PI3K−Akt signaling pathway
Proteoglycans in cancer
AGE−RAGE signaling pathway in diabetic complications
Amoebiasis
Relaxin signaling pathway
Vascular smooth muscle contraction
TGF−beta signaling pathway
Platelet activation
Human papillomavirus infection
Apelin signaling pathway
Hypertrophic cardiomyopathy
Dilated cardiomyopathy
Diabetic cardiomyopathy
Calcium signaling pathway
Fluid shear stress and atherosclerosis
Leukocyte transendothelial migration
Complement and coagulation cascades
Salivary secretion
Small cell lung cancer
Renin secretion
Rap1 signaling pathway
Oxytocin signaling pathway
Rheumatoid arthritis
cGMP−PKG signaling pathway
Nicotinate and nicotinamide metabolism
Circadian entrainment
Regulation of actin cytoskeleton
pvalue
0.005
0.010
0.015
0.020
0 5 10 15 20
Count
C
Blue
Turquoise
Green
DCN
ITGA1
COL11A1
COL4A1
POSTN
COL4A2
COL3A1
FN1
COL6A2
COL1A2
SPARC
COL1A1
COL6A3
COL5A1
MMP2
COL6A1
COL5A2
COL5A3
LUM
PCOLCE
ACTA2
CALD1
CTGF
THY1
SDC2
CD34
FMOD
ADAMTS4
TGFB3
BGN
ASPN
ACAN
SULF1
CDH11
FKBP10
COL12A1
CD248
COL14A1
PDGFRB
FLT4
FSTL1
NRP1
TNS1
PGF
ACTN1
KDR
CD24
COL18A1
FAP
NID1
MGP
SFRP2
LAMA4
VIM
LAMB2
CD163
NID2
TIMP2
CDH5
TIMP3
COL15A1
MMP3
VCAN
A2M
IGFBP4
MMP1
IGF2
MMP11
THBS1
TIMP1
ADAMTS2
SERPINE1
FBN1
VWF
DDR2
LOX
ITGA5
LOXL2
MYLK
NT5E
JAM3

Fig. s3

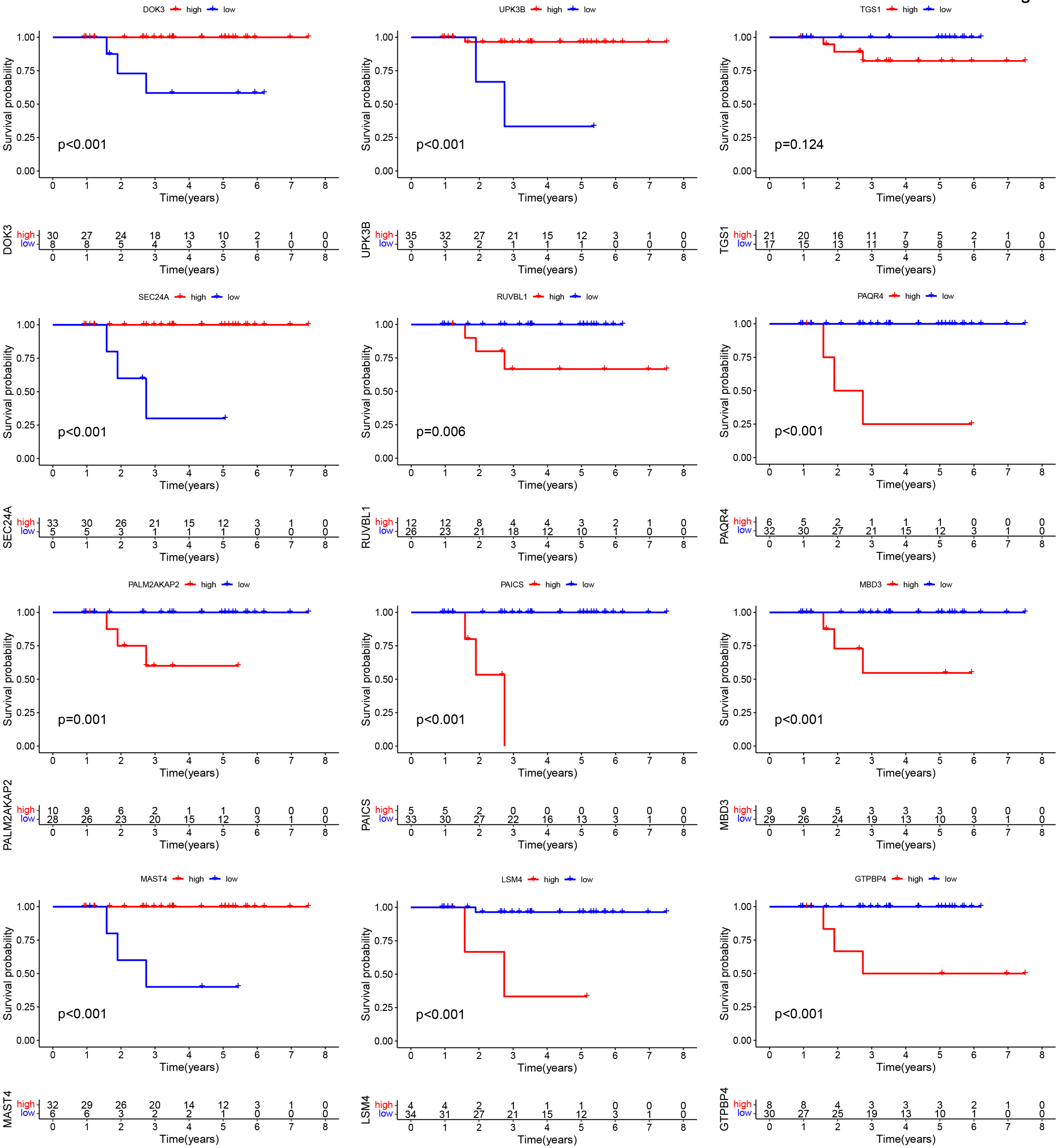

DOK3 high low
p<0.001
high 30 27 24 18 13 10 2 1 0
low 8 8 5 4 3 3 1 0 0
UPK3B high low
p<0.001
high 35 32 27 21 15 12 3 1 0
low 3 3 2 1 1 1 0 0 0
TGS1 high low
p=0.124
high 21 20 16 11 7 5 2 1 0
low 17 15 13 11 9 8 1 0 0
SEC24A high low
p<0.001
high 33 30 26 21 15 12 3 1 0
low 5 5 3 1 1 1 0 0 0
RUVBL1 high low
p=0.006
high 12 12 8 4 4 3 2 1 0
low 26 23 21 18 12 10 1 0 0
PAQR4 high low
p<0.001
high 6 5 2 1 1 1 0 0 0
low 32 30 27 21 15 12 3 1 0
PALM2AKAP2 high low
p=0.001
high 10 9 6 2 1 1 0 0 0
low 28 26 23 20 15 12 3 1 0
PAICS high low
p<0.001
high 5 5 2 0 0 0 0 0 0
low 33 30 27 22 16 13 3 1 0
MBD3 high low
p<0.001
high 9 9 5 3 3 3 0 0 0
low 29 26 24 19 13 10 3 1 0
MAST4 high low
p<0.001
high 32 29 26 20 14 12 3 1 0
low 6 6 3 2 2 1 0 0 0
LSM4 high low
p<0.001
high 4 4 2 1 1 1 0 0 0
low 34 31 27 21 15 12 3 1 0
GTPBP4 high low
p<0.001
high 8 8 4 3 3 3 2 1 0
low 30 27 25 19 13 10 1 0 0
Survival probability
Time(years)

Fig. s4

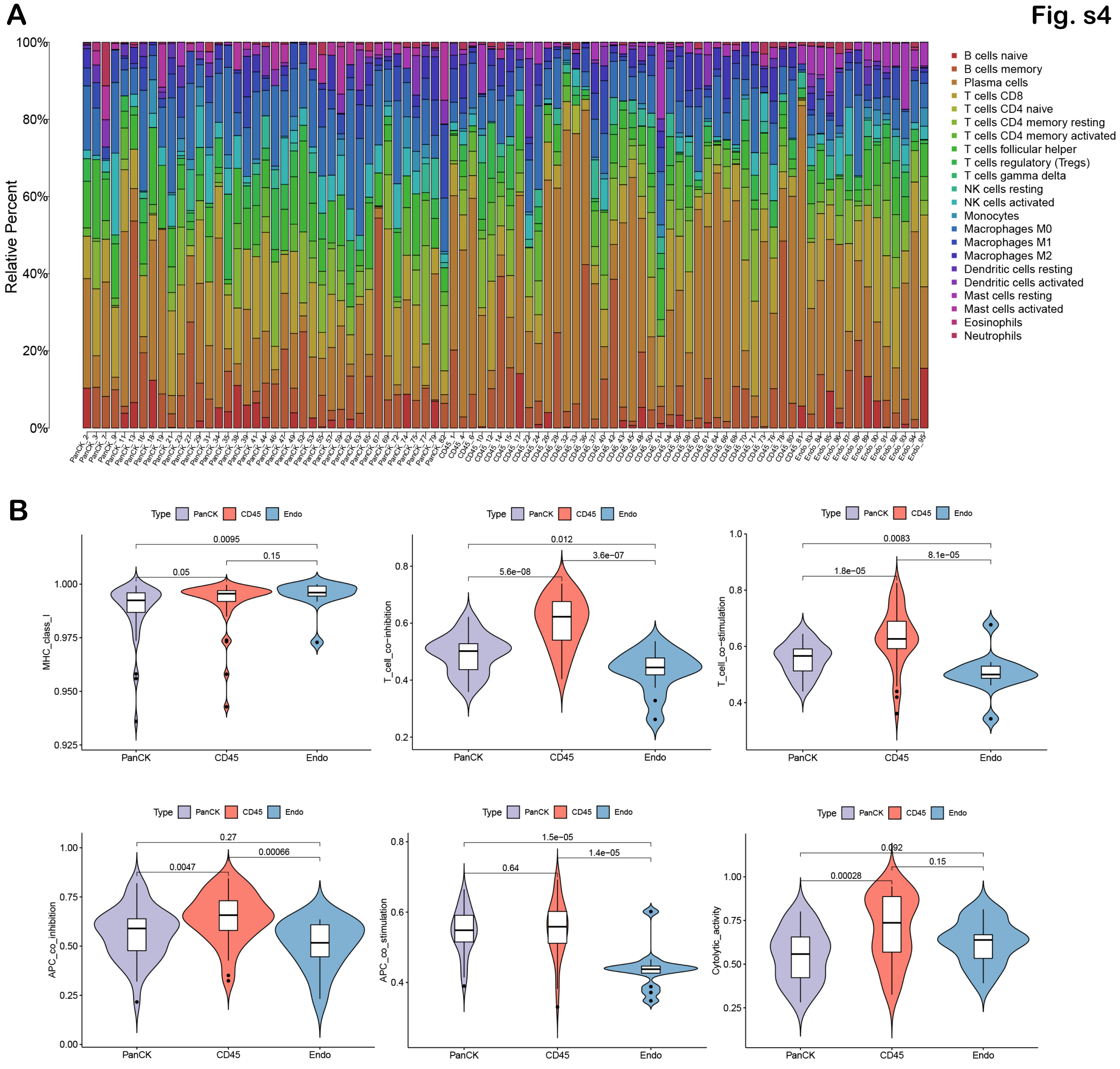
A
Relative Percent
100%
80%
60%
40%
20%
0%
B cells naive
B cells memory
Plasma cells
T cells CD8
T cells CD4 naive
T cells CD4 memory resting
T cells CD4 memory activated
T cells follicular helper
T cells regulatory (Tregs)
T cells gamma delta
NK cells resting
NK cells activated
Monocytes
Macrophages M0
Macrophages M1
Macrophages M2
Dendritic cells resting
Dendritic cells activated
Mast cells resting
Mast cells activated
Eosinophils
Neutrophils
B
Type PanCK CD45 Endo
MHC_class_I
0.0095
0.15
0.05
T_cell_co−inhibition
0.012
5.6e−08
3.6e−07
T_cell_co−stimulation
0.0083
1.8e−05
8.1e−05
APC_co_inhibition
0.27
0.0047
0.00066
APC_co_stimulation
1.5e−05
0.64
1.4e−05
Cytolytic_activity
0.092
0.00028
0.15
PanCK
CD45
Endo

Fig. s5

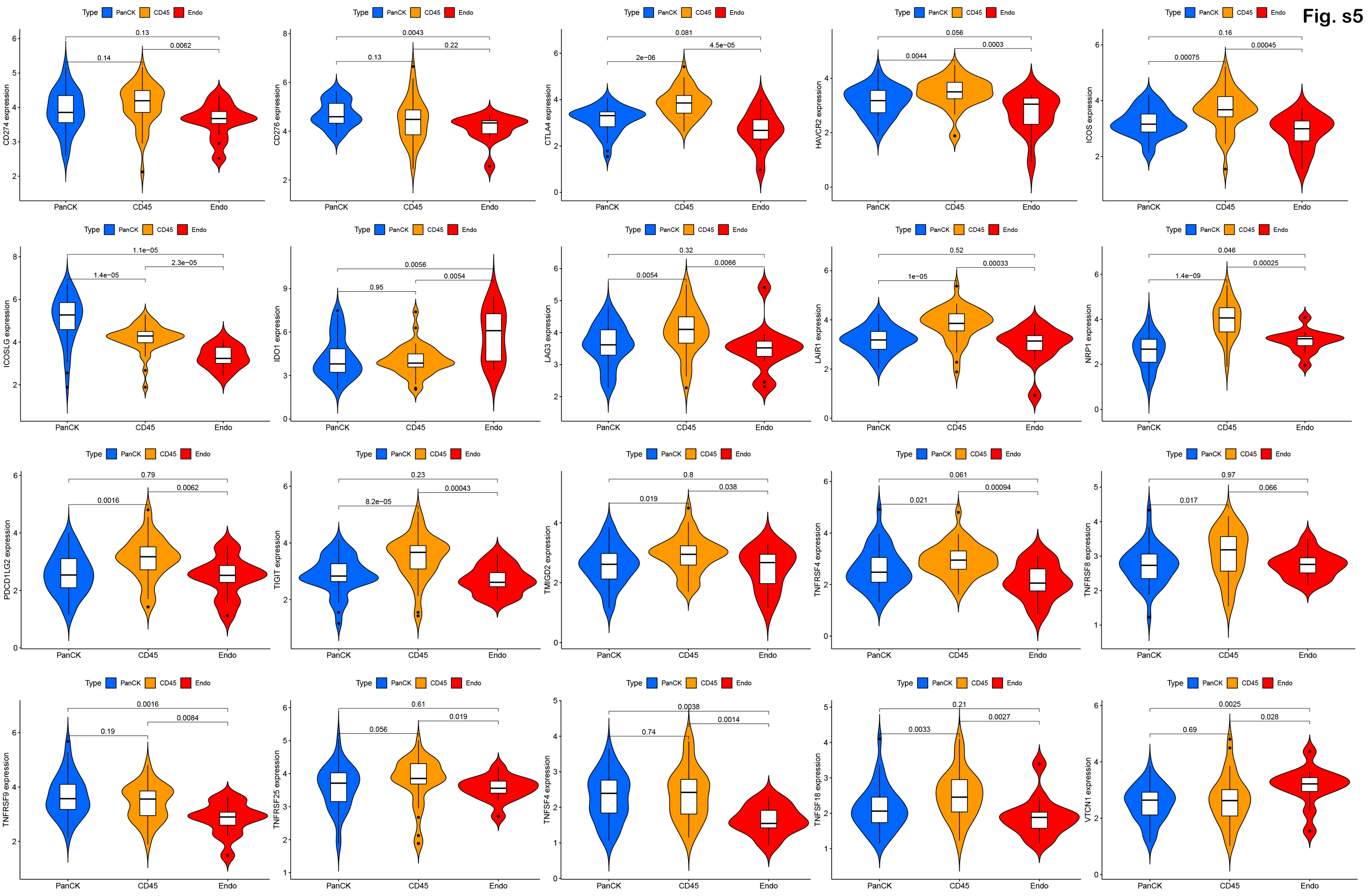
Type PanCK CD45 Endo
CD274 expression
0.13
0.14
0.0062
CD276 expression
0.0043
0.13
0.22
CTLA4 expression
0.081
2e−06
4.5e−05
HAVCR2 expression
0.056
0.0044
0.0003
ICOS expression
0.16
0.00075
0.00045
ICOSLG expression
1.1e−05
1.4e−05
2.3e−05
IDO1 expression
0.0056
0.95
0.0054
LAG3 expression
0.32
0.0054
0.0066
LAIR1 expression
0.52
1e−05
0.00033
NRP1 expression
0.046
1.4e−09
0.00025
PDCD1LG2 expression
0.79
0.0016
0.0062
TIGIT expression
0.23
8.2e−05
0.00043
TMIGD2 expression
0.8
0.019
0.038
TNFRSF4 expression
0.061
0.021
0.00094
TNFRSF8 expression
0.97
0.017
0.066
TNFRSF9 expression
0.0016
0.19
0.0084
TNFRSF25 expression
0.61
0.056
0.019
TNFSF4 expression
0.0038
0.74
0.0014
TNFSF18 expression
0.21
0.0033
0.0027
VTCN1 expression
0.0025
0.69
0.028
PanCK CD45 Endo

Fig. s6

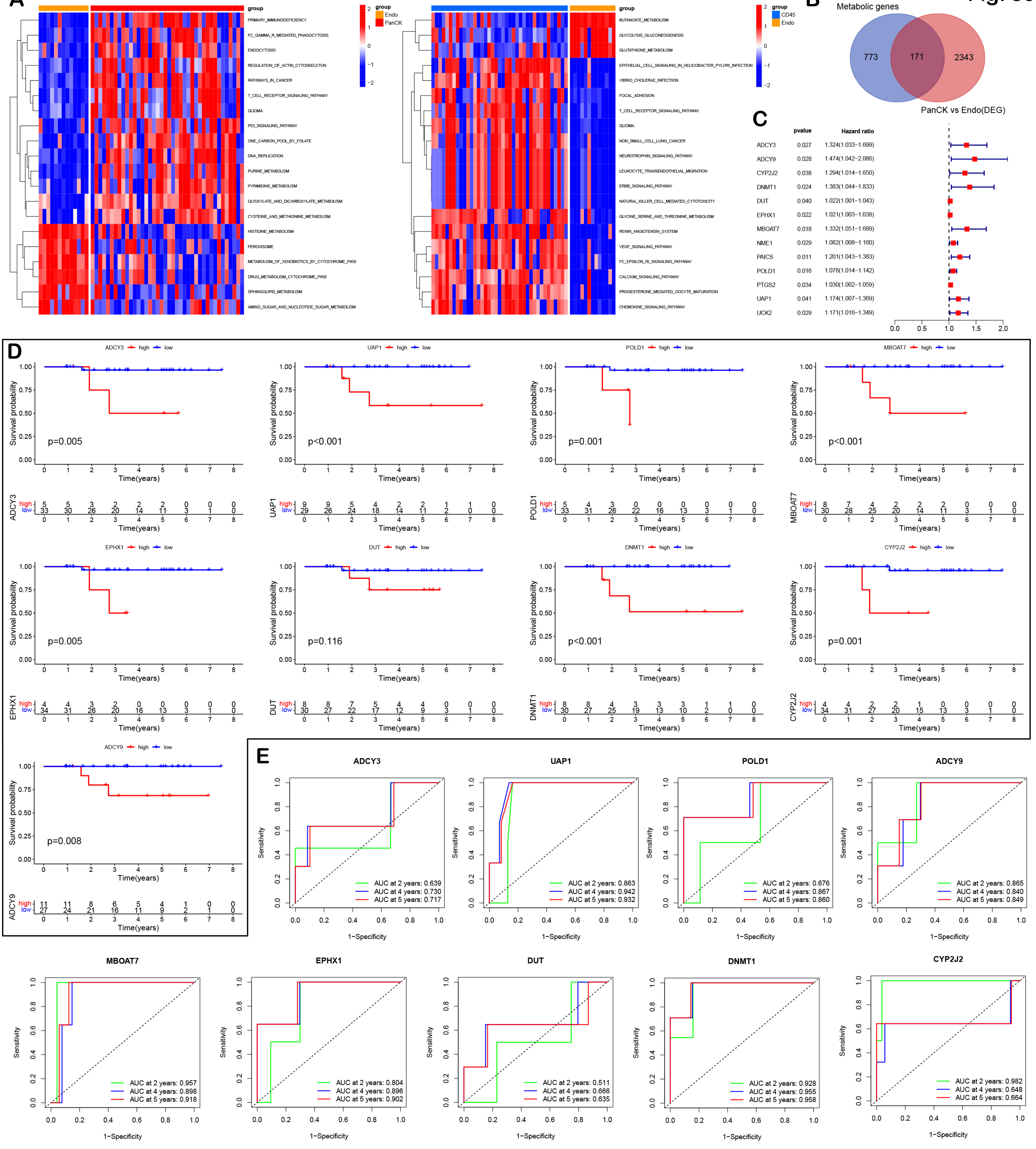

A
group
Endo
PanCK
PRIMARY_IMMUNODEFICIENCY
FC_GAMMA_R_MEDIATED_PHAGOCYTOSIS
ENDOCYTOSIS
REGULATION_OF_ACTIN_CYTOSKELETON
PATHWAYS_IN_CANCER
T_CELL_RECEPTOR_SIGNALING_PATHWAY
GLIOMA
P53_SIGNALING_PATHWAY
ONE_CARBON_POOL_BY_FOLATE
DNA_REPLICATION
PURINE_METABOLISM
PYRIMIDINE_METABOLISM
GLYOXYLATE_AND_DICARBOXYLATE_METABOLISM
CYSTEINE_AND_METHIONINE_METABOLISM
HISTIDINE_METABOLISM
PEROXISOME
METABOLISM_OF_XENOBIOTICS_BY_CYTOCHROME_P450
DRUG_METABOLISM_CYTOCHROME_P450
SPHINGOLIPID_METABOLISM
AMINO_SUGAR_AND_NUCLEOTIDE_SUGAR_METABOLISM
group
CD45
Endo
BUTANOATE_METABOLISM
GLYCOLYSIS_GLUCONEOGENESIS
GLUTATHIONE_METABOLISM
EPITHELIAL_CELL_SIGNALING_IN_HELICOBACTER_PYLORI_INFECTION
VIBRIO_CHOLERAE_INFECTION
FOCAL_ADHESION
T_CELL_RECEPTOR_SIGNALING_PATHWAY
GLIOMA
NON_SMALL_CELL_LUNG_CANCER
NEUROTROPHIN_SIGNALING_PATHWAY
LEUKOCYTE_TRANSENDOTHELIAL_MIGRATION
ERBB_SIGNALING_PATHWAY
NATURAL_KILLER_CELL_MEDIATED_CYTOTOXICITY
GLYCINE_SERINE_AND_THREONINE_METABOLISM
RENIN_ANGIOTENSIN_SYSTEM
VEGF_SIGNALING_PATHWAY
FC_EPSILON_RI_SIGNALING_PATHWAY
CALCIUM_SIGNALING_PATHWAY
PROGESTERONE_MEDIATED_OOCYTE_MATURATION
CHEMOKINE_SIGNALING_PATHWAY
B
Metabolic genes
773
171
2343
PanCK vs Endo(DEG)
C
pvalue Hazard ratio
ADCY3 0.027 1.324(1.033−1.699)
ADCY9 0.028 1.474(1.042−2.086)
CYP2J2 0.038 1.294(1.014−1.650)
DNMT1 0.024 1.383(1.044−1.833)
DUT 0.040 1.022(1.001−1.043)
EPHX1 0.022 1.021(1.003−1.038)
MBOAT7 0.018 1.332(1.051−1.689)
NME1 0.029 1.082(1.008−1.160)
PAICS 0.011 1.201(1.043−1.383)
POLD1 0.016 1.076(1.014−1.142)
PTGS2 0.034 1.030(1.002−1.059)
UAP1 0.041 1.174(1.007−1.369)
UCK2 0.029 1.171(1.016−1.349)
D
Survival probability
Time(years)
high low
ADCY3 p=0.005
UAP1 p<0.001
POLD1 p=0.001
MBOAT7 p<0.001
EPHX1 p=0.005
DUT p=0.116
DNMT1 p<0.001
CYP2J2 p=0.001
ADCY9 p=0.008
E
Sensitivity
1−Specificity
ADCY3
AUC at 2 years: 0.639
AUC at 4 years: 0.730
AUC at 5 years: 0.717
UAP1
AUC at 2 years: 0.863
AUC at 4 years: 0.942
AUC at 5 years: 0.932
POLD1
AUC at 2 years: 0.676
AUC at 4 years: 0.867
AUC at 5 years: 0.860
ADCY9
AUC at 2 years: 0.865
AUC at 4 years: 0.840
AUC at 5 years: 0.849
MBOAT7
AUC at 2 years: 0.957
AUC at 4 years: 0.898
AUC at 5 years: 0.918
EPHX1
AUC at 2 years: 0.804
AUC at 4 years: 0.896
AUC at 5 years: 0.902
DUT
AUC at 2 years: 0.511
AUC at 4 years: 0.666
AUC at 5 years: 0.635
DNMT1
AUC at 2 years: 0.928
AUC at 4 years: 0.955
AUC at 5 years: 0.958
CYP2J2
AUC at 2 years: 0.982
AUC at 4 years: 0.648
AUC at 5 years: 0.664

Supplementary Table 1. Characteristics of NPC samples in the DSP study

Characteristics	No. of samples (41)
Gender(male/female)	30/11
Age (<50/≥50)	29/12
Recurrence (yes/no)	5/36
Survival state (dead/alive)	4/37
Stage	
Stage I	19
Stage II	18
Stage III	1
Stage IV	3
EBER-ISH (-/+)	2/39

Supplementary Table 2. Characteristics of NPC samples in the validation cohort

Characteristics	No. of samples (121)
Gender(male/female)	77/44
Age (<50/⩾50)	46/75
Recurrence (yes/no)	52/69
Survival state (dead/alive)	29/92
T	
T1	45
T2	44
T3	29
T4	3
N	
N0	84
N1	35
N2	2
M	
M0	102
M1	19
EBV-VCA (+)	121
